# Supplementary material for: Distributed multi-parameter quantum metrology with a superconducting quantum network
Source: Nat Commun. 2026 Jan 20;17:1825. doi: 10.1038/s41467-026-68535-9 (PMC12921027; doi:10.1038/s41467-026-68535-9)
Supplement: Supplementary file 1 — Supplementary Information [file 41467_2026_68535_MOESM1_ESM.pdf]

Supplementary Information for “Distributed multi-parameter quantum metrology with a superconducting quantum network”

Jiajian Zhang,[1,*] Lingna Wang,[2,*] Yong-Ju Hai,[1,*] Jiawei Zhang,[3,1,*] Ji Chu,[1] Ji Jiang,[1] Wenhui Huang,[1] Yongqi Liang,[3,1] Jiawei Qiu,[1] Xuandong Sun,[3,1] Ziyu Tao,[1] Libo Zhang,[3,1] Yuxuan Zhou,[1] Yuanzhen Chen,[3] Weijie Guo,[1] Xiayu Linpeng,[1] Song Liu,[1,4] Wenhui Ren,[1] Youpeng Zhong,[1,4,†] Jingjing Niu,[1,4,‡] Haidong Yuan,[2,§] and Dapeng Yu[1,4]

[1]*International Quantum Academy, Shenzhen 518048, China*
[2]*Department of Mechanical and Automation Engineering, The Chinese University of Hong Kong, Shatin, Hong Kong*
[3]*Southern University of Science and Technology, Shenzhen 518055, China*
[4]*Shenzhen Branch, Hefei National Laboratory, Shenzhen 518048, China*

CONTENTS

Supplementary Note 1 – Theoretical analysis **2**
1.1 General process of quantum metrology 2
1.2 Sensing of a remote vector field 3
1.3 Sensing of the gradients between vector fields 6
1.3.1 Strategy with non-local entanglement 6
1.3.2 Strategy with local entanglement 10
1.3.3 Additional strategy and comparison 13

Supplementary Note 2 – Experimental implementation **16**
2.1 Device information 16
2.2 Gate performance 18
2.3 Gate set calibration 18
2.4 Implementation of distributed sensing in quantum circuits 19

Supplementary Note 3 – Extended data **24**
3.1 Extended data for sensing of remote vector fields 24
3.2 Extended data for distributed sensing of vector field gradient 26
3.3 The influence of noise 27

Supplementary References **29**

[*] These authors contributed equally to this work.
[†] zhongyoupeng@iqasz.cn
[‡] niujj@iqasz.cn
[§] hdyuan@mae.cuhk.edu.hk

Supplementary Note 1 – Theoretical analysis

1.1 General process of quantum metrology

The primary objective of quantum metrology is to precisely estimate unknown physical quantities by utilizing quantum resources, such as quantum entanglement. The general process of quantum metrology includes the following steps: state preparation, parameter encoding, measurement, and estimation. The probe state ρ_0 evolves under the given dynamics, which depend on the unknown parameters $\mathbf{x} = (x_1, \cdots, x_n)$, resulting in the encoded state $\rho(\mathbf{x})$. To extract information about the parameters $\mathbf{x}$, we perform a set of positive operator-valued measures (POVMs), represented as $\{\Pi_i\}$, on the state $\rho(\mathbf{x})$, obtaining a set of probability distributions $P_i(\mathbf{x})$, where $P_i(\mathbf{x})$ is the probability of obtaining the measurement result i. Finally, we construct the estimators $\mathbf{x}_{\text{est}} = (x_{1_{\text{est}}}, \cdots, x_{n_{\text{est}}})$ based on the probabilities of the measurement outcomes. For multi-parameter quantum estimating, the performance of locally unbiased estimators is quantified by the covariance matrix, where the jk-th element gives

$$[\text{Cov}(\mathbf{x}_{\text{est}})]_{jk} = E\left[(x_{j_{\text{est}}} - x_j)(x_{k_{\text{est}}} - x_k)\right]. \tag{1}$$

The estimation precision for multiple parameters is quantified by the sum of variances, which corresponds to the sum of the diagonal terms of the covariance matrix. The covariance matrix is lower bounded by

$$\text{Cov}(\mathbf{x}_{\text{est}}) \geq \frac{1}{n} F_C^{-1} \tag{2}$$

which is known as the Cramér-Rao bound. Here n is the number of measurement repetitions, and F_C is the Fisher information matrix (FIM) with jk-th element calculated as follows:

$$[F_C]_{x_j x_k} = \sum_i \frac{1}{P_i(\mathbf{x})} \left(\frac{\partial P_i(\mathbf{x})}{\partial x_j}\right) \left(\frac{\partial P_i(\mathbf{x})}{\partial x_k}\right) \tag{3}$$

The Cramér-Rao bound is achievable for a large number of repetitions by using the maximum likelihood estimator (MLE). The quantum Cramér-Rao bound (QCRB) further constrains the covariance matrix:

$$\text{Cov}(\mathbf{x}_{\text{est}}) \geq \frac{1}{n} F_C^{-1} \geq \frac{1}{n} F_Q^{-1}. \tag{4}$$

Here F_Q is the quantum Fisher information matrix (QFIM). For parameters encoded in pure states $|\psi_{\mathbf{x}}\rangle$, F_Q can be expressed as:

$$[F_Q]_{x_j x_k} = 4\,\text{Re}\left(\langle \partial_j \psi_{\mathbf{x}} | \partial_k \psi_{\mathbf{x}} \rangle - \langle \partial_j \psi_{\mathbf{x}} | \psi_{\mathbf{x}} \rangle \langle \psi_{\mathbf{x}} | \partial_k \psi_{\mathbf{x}} \rangle\right). \tag{5}$$

More specifically, consider a pure probe state $|\psi_0\rangle$ undergoes a unitary process $U_{\mathbf{x}}$, the encoded state $|\psi_{\mathbf{x}}\rangle = U_{\mathbf{x}} |\psi_0\rangle$. Define the generator of $U_{\mathbf{x}}$ corresponding to unknown parameter x_j as

$$h_{x_j} = i U_{\mathbf{x}}^{\dagger} \left(\partial_{x_j} U_{\mathbf{x}}\right). \tag{6}$$

Then, the quantum Fisher information matrix in Supplementary Equation 5 can be expressed in terms of the generators as

$$[F_Q]_{x_j x_k} = 2\langle\psi_0|\{h_{x_j}, h_{x_k}\}|\psi_0\rangle - 4\langle\psi_0|h_{x_j}|\psi_0\rangle\langle\psi_0|h_{x_k}|\psi_0\rangle. \tag{7}$$

Here $\{\cdot,\cdot\}$ denotes the anti-commutator. According to QCRB, the precision of estimating multiple parameters is lower bound by $n\mathrm{Tr}(\mathrm{Cov}(\mathbf{x}_{\mathrm{est}})) \geq \mathrm{Tr}(F_C^{-1}) \geq \mathrm{Tr}(F_Q^{-1})$. Hence, finding the maximal QFIM and the optimal measurement that saturates the QCRB leads to the ultimate precision of estimation. For multi-parameter quantum estimation, the necessary and sufficient condition for saturating the quantum Cramér-Rao bound in pure states is the weak commutativity condition, which is

$$\mathrm{Im}\left[\langle\partial_{x_j}\psi_{\mathbf{x}}|\partial_{x_k}\psi_{\mathbf{x}}\rangle\right] = 0, \forall x_j, x_k. \tag{8}$$

To achieve the best precision for estimating unknown parameters $\mathbf{x}$, it is crucial to optimize every step of the process: the initial state, the controls during the evolution, and the final measurement. In the control-enhanced sequential scheme, the total system evolution is described by $U_N = (U_c U_{\mathbf{x}})^N$, where $U_{\mathbf{x}}$ represents the system dynamics over time T, and U_c is a control operation applied after each cycle. The optimal strategy, as derived in [1], employs the control $U_c = U_{\mathbf{x}}^\dagger$. This choice leads to a quadratic enhancement in precision, scaling the quantum Fisher information matrix as $N^2 F_Q$, where F_Q is the QFIM for a single cycle ($N = 1$). In the following analysis, we will first obtain the maximal F_Q for a single cycle. The corresponding QFIM for a multi-cycle strategy with optimal control is then obtained by scaling F_Q by N^2. We note that the maximal F_Q for a single cycle is equivalent to the maximal QFIM achievable under the dynamics $U_{\mathbf{x}}$ without the control. This is because a single control operation applied only at the end of the evolution cannot improve the QFIM.

1.2 Sensing of a remote vector field

We consider the estimation of three components of a remote vector field, described in spherical coordinates (B, θ, ϕ) as $\mathbf{B} = (B\sin\theta\cos\phi, B\sin\theta\sin\phi, B\cos\theta)$, instead of in Cartesian coordinates $\mathbf{B} = (B_x, B_y, B_z)$. Estimating the vector field components $\mathbf{B} = (B_x, B_y, B_z)$ thus corresponds to simultaneously estimating the parameters $\mathbf{x} = (B, \theta, \phi)$. For each sensor qubit at time T, the evolution can be represented by $U_s = e^{-i\mathbf{B}\cdot\boldsymbol{\sigma}T} = e^{-iBT\boldsymbol{n}\cdot\boldsymbol{\sigma}}$ with $\boldsymbol{n} = (\sin\theta\cos\phi, \sin\theta\sin\phi, \cos\theta)$. The generator for $x_j \in \{B, \theta, \phi\}$ is given by

$$\begin{aligned} h_B &= c_B \boldsymbol{n}_B \cdot \boldsymbol{\sigma} \\ h_\theta &= c_\theta \boldsymbol{n}_\theta \cdot \boldsymbol{\sigma} \\ h_\phi &= c_\phi \boldsymbol{n}_\phi \cdot \boldsymbol{\sigma} \end{aligned} \tag{9}$$

with

$$c_B = T, \quad c_\theta = \sin(BT), \quad c_\phi = \sin(BT)\sin\theta, \tag{10}$$

$$\begin{aligned}
\boldsymbol{n}_B &= \boldsymbol{n} = (\sin\theta\cos\phi, \sin\theta\sin\phi, \cos\theta),\\
\boldsymbol{n}_\theta &= \cos(BT)\boldsymbol{n}_1 - \sin(BT)\boldsymbol{n}_2,\\
\boldsymbol{n}_\phi &= \sin(BT)\boldsymbol{n}_1 + \cos(BT)\boldsymbol{n}_2,
\end{aligned} \tag{11}$$

where $\boldsymbol{n}_1 = \partial_\theta \boldsymbol{n} = (\cos\theta\cos\phi, \cos\theta\sin\phi, -\sin\theta)$, $\boldsymbol{n}_2 = \boldsymbol{n}\times\boldsymbol{n}_1 = (-\sin\phi, \cos\phi, 0)$. It is easy to verify that $\boldsymbol{n}$, $\boldsymbol{n}_1$, $\boldsymbol{n}_2$ are orthogonal to each other, since there exists a unitary transformation $U_r = e^{i\frac{BT}{2}\boldsymbol{n}\cdot\boldsymbol{\sigma}} e^{-i\frac{\phi}{2}\sigma_z} e^{-i\frac{\theta}{2}\sigma_y}$ such that

$$\boldsymbol{n}_B\cdot\boldsymbol{\sigma} = U_r\sigma_z U_r^\dagger, \quad \boldsymbol{n}_\theta\cdot\boldsymbol{\sigma} = U_r\sigma_x U_r^\dagger, \quad \boldsymbol{n}_\phi\cdot\boldsymbol{\sigma} = U_r\sigma_y U_r^\dagger. \tag{12}$$

Assume the initial probe state is $|\psi_{SA}\rangle$, where the ancilla system is introduced. Then using Supplementary Equation 7, we obtain the QFIM as

$$F_Q = 4\begin{pmatrix} T^2 - (\mathrm{Tr}(\rho_S h_B))^2 & -\mathrm{Tr}(\rho_S h_B)\,\mathrm{Tr}(\rho_S h_\theta) & -\mathrm{Tr}(\rho_S h_B)\,\mathrm{Tr}(\rho_S h_\phi) \\ -\mathrm{Tr}(\rho_S h_B)\,\mathrm{Tr}(\rho_S h_\theta) & \sin^2(BT) - (\mathrm{Tr}(\rho_S h_\theta))^2 & -\mathrm{Tr}(\rho_S h_\theta)\,\mathrm{Tr}(\rho_S h_\phi) \\ -\mathrm{Tr}(\rho_S h_B)\,\mathrm{Tr}(\rho_S h_\phi) & -\mathrm{Tr}(\rho_S h_\theta)\,\mathrm{Tr}(\rho_S h_\phi) & \sin^2(BT)\sin^2\theta - (\mathrm{Tr}(\rho_S h_\theta))^2 \end{pmatrix} \tag{13}$$

where $\rho_S = \mathrm{Tr}_A(|\psi_{SA}\rangle\langle\psi_{SA}|)$ denotes the reduced state by tracing out the ancilla system. The maximal QFIM, denoted as $F_Q^{\max}$, is achieved when $\rho_S = \frac{1}{2}I$ with

$$F_Q^{\max} = 4\begin{pmatrix} T^2 & 0 & 0 \\ 0 & \sin^2(BT) & 0 \\ 0 & 0 & \sin^2(BT)\sin^2\theta \end{pmatrix}. \tag{14}$$

Here $F_Q^{\max}$ is maximal in the sense that $F_Q^{max} - F_Q \geq 0$ for any other F_Q. The optimal state can be chosen as any pure state $|\psi_{SA}\rangle$ with the reduced state $\rho_S = \frac{1}{2}I$. This can be any maximally entangled state, in particular, it can be chosen as $|\psi_{SA}\rangle = \frac{1}{\sqrt{2}}(|00\rangle + |11\rangle)$.

Next we show the projective measurement in the Bell basis saturates the quantum Cramér-Rao bound. The projective measurement in the Bell basis is given by

$$M_{00} = \left|\Phi^+\right\rangle\left\langle\Phi^+\right|, \quad M_{01} = \left|\Phi^-\right\rangle\left\langle\Phi^-\right|, \quad M_{10} = \left|\Psi^+\right\rangle\left\langle\Psi^+\right|, \quad M_{11} = \left|\Psi^-\right\rangle\left\langle\Psi^-\right|, \tag{15}$$

where

$$\begin{aligned}
\left|\Phi^+\right\rangle &= \frac{1}{\sqrt{2}}(|00\rangle + |11\rangle), \quad \left|\Phi^-\right\rangle = \frac{1}{\sqrt{2}}(|00\rangle - |11\rangle),\\
\left|\Psi^+\right\rangle &= \frac{1}{\sqrt{2}}(|01\rangle + |10\rangle), \quad \left|\Psi^-\right\rangle = \frac{1}{\sqrt{2}}(|01\rangle - |10\rangle),
\end{aligned} \tag{16}$$

are the Bell states. Under this measurement, the probabilities of the measurement outcomes are given by

$$\begin{aligned}
P_{00} &= \mathrm{Tr}(\rho(B,\theta,\phi)M_{00}) = \cos^2(BT),\\
P_{01} &= \mathrm{Tr}(\rho(B,\theta,\phi)M_{01}) = \sin^2(BT)\cos^2\theta,\\
P_{10} &= \mathrm{Tr}(\rho(B,\theta,\phi)M_{10}) = \sin^2(BT)\sin^2\theta\cos^2\phi,\\
P_{11} &= \mathrm{Tr}(\rho(B,\theta,\phi)M_{11}) = \sin^2(BT)\sin^2\theta\sin^2\phi,
\end{aligned} \tag{17}$$

where $\rho(B,\theta,\phi) = (U_s \otimes I)|\psi_{SA}\rangle\langle\psi_{SA}|(U_s^\dagger \otimes I)$ is the evolved state. From these probabilities, we can obtain the classical Fisher information matrix via Supplementary Equation 3 as

$$F_C = 4 \begin{pmatrix} T^2 & 0 & 0 \\ 0 & \sin^2(BT) & 0 \\ 0 & 0 & \sin^2(BT)\sin^2\theta \end{pmatrix}. \tag{18}$$

This is identical to the quantum Fisher information matrix $F_Q^{\max}$, which verifies the projective measurement in the Bell basis saturates the quantum Cramér-Rao bound. The quantum Cramér-Rao bound $\mathrm{Cov}(\mathbf{x}_{\mathrm{est}}) \geq F_Q^{-1}$(here we neglect the repetition n, which is a classical effect and the same for all schemes) is thus achievable, which leads to the precision for the three parameters in the spherical coordinates as

$$\delta B_{\mathrm{est}}^2 \geq \frac{1}{4T^2}, \quad \delta\theta_{\mathrm{est}}^2 \geq \frac{1}{4\sin^2(BT)}, \quad \delta\phi_{\mathrm{est}}^2 \geq \frac{1}{4\sin^2(BT)\sin^2\theta}. \tag{19}$$

In the Euclidean coordinates, this gives an achievable bound on the total variances as

$$\begin{aligned} \delta B_{x_{\mathrm{est}}}^2 + \delta B_{y_{\mathrm{est}}}^2 + \delta B_{z_{\mathrm{est}}}^2 &= \delta B_{\mathrm{est}}^2 + B^2\delta\theta_{\mathrm{est}}^2 + B^2\sin^2\theta\delta\phi_{\mathrm{est}}^2 \\ &\geq \frac{1}{4T^2} + \frac{B^2}{2\sin^2(BT)}. \end{aligned} \tag{20}$$

For N cycles, the maximal quantum Fisher information matrix is given by $N^2 F_Q^{\max}$. The corresponding precisions are

$$\delta B_{\mathrm{est}}^2 \geq \frac{1}{4N^2T^2}, \quad \delta\theta_{\mathrm{est}}^2 \geq \frac{1}{4N^2\sin^2(BT)}, \quad \delta\phi_{\mathrm{est}}^2 \geq \frac{1}{4N^2\sin^2(BT)\sin^2\theta}. \tag{21}$$

In comparison, the individual strategy, referred to as the "classical individual measurement" protocol in [2], involves measuring the system immediately after one cycle. The process is then repeated $N/3$ times for estimating one parameter—N operators total for estimating all three parameters. The resulting precisions are given by:

$$\delta B_{\mathrm{est}}^2 \geq \frac{3}{4NT^2}, \quad \delta\theta_{\mathrm{est}}^2 \geq \frac{3}{4N\sin^2(BT)}, \quad \delta\phi_{\mathrm{est}}^2 \geq \frac{3}{4N\sin^2(BT)\sin^2\theta},$$

which are taken as the standard quantum limit.

When the N operators act sequentially on the system qubit without the control, the total evolution is given by $[U_s(\mathbf{x})]^N = e^{-iN\mathbf{B}\cdot\boldsymbol{\sigma}T}$, here $\mathbf{x} = (B,\theta,\phi)$ and $\mathbf{B} = (B\sin\theta\cos\phi, B\sin\theta\sin\phi, B\cos\theta)$. In this case the maximal quantum Fisher information matrix is

$$F_Q^{\max} = 4 \begin{pmatrix} N^2T^2 & 0 & 0 \\ 0 & \sin^2(NBT) & 0 \\ 0 & 0 & \sin^2(NBT)\sin^2\theta \end{pmatrix}, \tag{22}$$

which gives

$$\delta B_{\mathrm{est}}^2 \geq \frac{1}{4N^2T^2}, \quad \delta\theta_{\mathrm{est}}^2 \geq \frac{1}{4\sin^2(NBT)}, \quad \delta\phi_{\mathrm{est}}^2 \geq \frac{1}{4\sin^2(NBT)\sin^2\theta}. \tag{23}$$

While this strategy improves the scaling for the estimation of the amplitude B, its performance for the estimation of θ and ϕ can degrade significantly and even become worse than the individual strategy. In particular, under the experimental condition used in our study, $BT = 3\pi/2$, the sine terms vanish when N is an even number, leading to diverging estimation errors for both θ and ϕ. For this reason, we do not include this strategy for the comparison in the main text.

1.3 Sensing of the gradients between vector fields

In this section, we consider the estimation of the gradients between two remote vector fields, expressed as $\nabla \mathbf{B} = \mathbf{B}_1 - \mathbf{B}_2 = (\nabla B_x, \nabla B_y, \nabla B_z)$. For each sensor qubit, the Hamiltonian H_j is given by $H_j = \mathbf{B}_j \cdot \boldsymbol{\sigma} = B_{jx}\sigma_x + B_{jy}\sigma_y + B_{jz}\sigma_z$, where $\mathbf{B}_j = (B_{jx}, B_{jy}, B_{jz})$ represents the vector field components and $\boldsymbol{\sigma} = (\sigma_x, \sigma_y, \sigma_z)$ denotes the spin vector. Alternatively, in spherical coordinates, the Hamiltonian can be represented as $H_j = B_j \boldsymbol{n}_j \cdot \boldsymbol{\sigma}$, where $\boldsymbol{n}_j = (\sin\theta_j \cos\phi_j, \sin\theta_j \sin\phi_j, \cos\theta_j)$ and $B_j = \sqrt{B_{jx}^2 + B_{jy}^2 + B_{jz}^2}$ represents the magnitude of the vector field. The evolution at time T can be represented by $U_{sj} = e^{-i\mathbf{B}_j \cdot \boldsymbol{\sigma} T} = e^{-iB_j \boldsymbol{n}_j \cdot \boldsymbol{\sigma} T}$ for $j = 1, 2$.

We compare the precision of two strategies for estimating a vector field's gradient. The first uses non-local entanglement to directly measure the gradient across the sensor network. The second employs only local entanglement, measuring the field independently at each point before calculating the difference. Our analysis confirms that non-local entanglement provides a distinct quantum advantage, offering superior precision for distributed gradient sensing.

1.3.1 Strategy with non-local entanglement

In this approach, we employ a 4-qubit non-local entangled state, $|\Psi_0\rangle = \frac{1}{\sqrt{2}}(|0011\rangle - |1100\rangle)$, as the probe state to directly estimate gradients along three directions. The first two and last two qubits are distributed to two separate sensor modules (denoted $\mathcal{B}$ and $\mathcal{C}$ in the main text) respectively. The system undergoes a total evolution described by $U_{\mathrm{S}} = U_{s1} \otimes U_{s1} \otimes U_{s2} \otimes U_{s2}$, which imparts information about the spatial gradients onto the evolved state $U_{\mathrm{S}}|\Psi_0\rangle$. The components of the vector fields at the two locations can be expressed in terms of their sum and gradient:

$$B_{1j} = \frac{\sum B_j + \nabla B_j}{2}, \quad B_{2j} = \frac{\sum B_j - \nabla B_j}{2}, \quad \text{for } j \in x, y, z, \tag{24}$$

where $\sum \mathbf{B} = \mathbf{B}_1 + \mathbf{B}_2 = (\sum B_x, \sum B_y, \sum B_z)$ and $\nabla \mathbf{B} = \mathbf{B}_1 - \mathbf{B}_2 = (\nabla B_x, \nabla B_y, \nabla B_z)$

To effectively estimate the gradients, it is also necessary to acquire information about the sum of the vector fields. This information is crucial for adaptively implementing control strategies. Such control strategies essentially reduce the problem of estimating general gradients $\nabla \mathbf{B}$ to estimating $\nabla \mathbf{B} = (0, 0, 0)$, indicating $\mathbf{B}_1 = \mathbf{B}_2 = \mathbf{B}$. This can be realized by adding compensation to the vector fields if the gradients are non-zero. Therefore, we benchmark the performance of our estimation

protocol at zero-gradient, and the comparison of different strategies will also be made under this assumption.

The quantum Fisher information matrix for the simultaneous estimation of the parameters $\mathbf{x} = (\nabla\mathbf{B}, \sum\mathbf{B})$ under the dynamics U_{S} is block-diagonal:

$$F_Q = \begin{pmatrix} F_- & \mathbf{0} \\ \mathbf{0} & F_+ \end{pmatrix}, \tag{25}$$

where F_- and F_+ are the QFIM submatrices for estimating the gradient $\nabla\mathbf{B} = (\nabla B_x, \nabla B_y, \nabla B_z)$ and the sum $\sum\mathbf{B} = (\sum B_x, \sum B_y, \sum B_z)$, respectively. The matrix F_- is given by

$$F_- = \begin{pmatrix} [F_Q]_{\nabla B_x \nabla B_x} & [F_Q]_{\nabla B_x \nabla B_y} & [F_Q]_{\nabla B_x \nabla B_z} \\ [F_Q]_{\nabla B_x \nabla B_y} & [F_Q]_{\nabla B_y \nabla B_y} & [F_Q]_{\nabla B_y \nabla B_z} \\ [F_Q]_{\nabla B_x \nabla B_z} & [F_Q]_{\nabla B_y \nabla B_z} & [F_Q]_{\nabla B_z \nabla B_z} \end{pmatrix}, \tag{26}$$

with its elements

$$\begin{aligned}
[F_Q]_{\nabla B_x \nabla B_x} =& \frac{4}{B^4}\left(B_x^2 T^2 (B^2 + 3B_z^2) + \sin^2(BT)(B_y^2 + B_z^2 + 6B_x B_y B_z T + 3B_y^2 \sin^2(BT))\right) \\
&+ \frac{3B_x B_z \sin(2BT)}{B^6}\left(-4BB_y \sin^2(BT) + B_x B_z(-4BT + \sin(2BT)\right), \\
[F_Q]_{\nabla B_y \nabla B_y} =& \frac{4}{B^4}\left(B_y^2 T^2 (B^2 + 3B_z^2) + \sin^2(BT)(B_x^2 + B_z^2 - 6B_x B_y B_z T + 3B_x^2 \sin^2(BT))\right) \\
&+ \frac{3B_y B_z \sin(2BT)}{B^6}\left(4BB_x \sin^2(BT) + B_y B_z(-4BT + \sin(2BT)\right) \\
[F_Q]_{\nabla B_z \nabla B_z} =& \frac{4}{B^4}\left(B_z^2 T^2 (B^2 + 3B_z^2) + \sin^2(BT)(B_x^2 + B_y^2)\right) \\
&+ \frac{3\sin(2BT)}{B^6}\left(4B(B_x^2 + B_y^2)B_z^2 T + (B_x^2 + B_y^2)^2 \sin(2BT)\right), \\
[F_Q]_{\nabla B_x \nabla B_y} =& \frac{4}{B^4}\left(B_x B_y T^2 (B^2 + 3B_z^2) - \sin^2(BT)(4B_x B_y + 3B_z T(B_x^2 - B_y^2))\right) \\
&+ \frac{3\sin(2BT)}{B^6}\left(2BB_z \sin^2(BT)(B_x^2 - B_y^2) + B_x B_y(-4BB_z^2 T + (B^2 + B_z^2)\sin(2BT))\right), \\
[F_Q]_{\nabla B_x \nabla B_z} =& \frac{4B_z}{B^4}\left(B_x T^2 (B^2 + 3B_z^2) - \sin^2(BT)(B_x - 3B_y B_z T)\right) \\
&+ \frac{3\sin(2BT)}{B^6}\left(2BB_y \sin^2(BT)(B_x^2 + B_y^2) + B_x B_z(2BT(B^2 - 2B_z^2) - (B^2 - B_z^2)\sin(2BT))\right), \\
[F_Q]_{\nabla B_y \nabla B_z} =& \frac{4B_z}{B^4}\left(B_y T^2 (B^2 + 3B_z^2) - \sin^2(BT)(B_y + 3B_x B_z T)\right) \\
&- \frac{3\sin(2BT)}{B^6}\left(2BB_x \sin^2(BT)(B_x^2 + B_y^2) - B_y B_z(2BT(B^2 - 2B_z^2) - (B^2 - B_z^2)\sin(2BT))\right).
\end{aligned} \tag{27}$$

Similarly, the matrix F_+ is given by

$$F_+ = \begin{pmatrix} [F_Q]_{\sum B_x \sum B_x} & [F_Q]_{\sum B_x \sum B_y} & [F_Q]_{\sum B_x \sum B_z} \\ [F_Q]_{\sum B_x \sum B_y} & [F_Q]_{\sum B_y \sum B_y} & [F_Q]_{\sum B_y \sum B_z} \\ [F_Q]_{\sum B_x \sum B_z} & [F_Q]_{\sum B_y \sum B_z} & [F_Q]_{\sum B_z \sum B_z} \end{pmatrix}, \tag{28}$$

with its elements

$$
\begin{aligned}
[F_Q]_{\sum B_x \sum B_x} =& \frac{4}{B^4}\left(B_x^2T^2(B_x^2+B_y^2)+B_z\sin^2(BT)(B_z-2B_xB_yT)\right)\\
&+\frac{\sin(2BT)}{B^6}\left(4BB_x^2B_z^2T+4BB_xB_yB_z\sin^2(BT)+(B^2B_y^2-B_x^2B_z^2)\sin(2BT)\right),\\
[F_Q]_{\sum B_y \sum B_y} =& \frac{4}{B^4}\left(B_y^2T^2(B_x^2+B_y^2)+B_z\sin^2(BT)(B_z+2B_xB_yT)\right)\\
&+\frac{\sin(2BT)}{B^6}\left(4BB_y^2B_z^2T-4BB_xB_yB_z\sin^2(BT)+(B^2B_x^2-B_y^2B_z^2)\sin(2BT)\right),\\
[F_Q]_{\sum B_z \sum B_z} =& \frac{4}{B^4}(B_x^2+B_y^2)(B_z^2T^2+\sin^2(BT))\\
&+\frac{\sin(2BT)}{B^6}(B_x^2+B_y^2)\left(4BB_z^2T+(B_x^2+B_y^2)\sin(2BT)\right)\\
[F_Q]_{\sum B_x \sum B_y} =& \frac{4T}{B^4}\left(B_xB_yT(B_x^2+B_y^2)+B_z\sin^2(BT)(B_x^2-B_y^2)\right)\\
&+\frac{\sin(2BT)}{B^6}\left(4BB_xB_yB_z^2T-2BB_z\sin^2(BT)(B_x^2-B_y^2)-B_xB_y\sin(2BT)(B^2+B_z^2)\right),\\
[F_Q]_{\sum B_x \sum B_z} =& \frac{4B_z}{B^4}\left(B_xT^2(B_x^2+B_y^2)-\sin^2(BT)(B_x+B_yB_zT)\right)\\
&-\frac{\sin(2BT)}{B^6}\left(2BB_xB_zT(B^2-2B_z^2)+(B_x^2+B_y^2)\left(2BB_y\sin^2(BT)-B_xB_z\sin(2BT)\right)\right),\\
[F_Q]_{\sum B_y \sum B_z} =& \frac{4B_z}{B^4}\left(B_yT^2(B_x^2+B_y^2)-\sin^2(BT)(B_y-B_xB_zT)\right)\\
&-\frac{\sin(2BT)}{B^6}\left(2BB_yB_zT(B^2-2B_z^2)-(B_x^2+B_y^2)\left(2BB_x\sin^2(BT)+B_yB_z\sin(2BT)\right)\right).
\end{aligned}
\tag{29}
$$

Due to the block-diagonal structure of the quantum Fisher information matrix, the precision of estimating the gradient components $\nabla\mathbf{B}$ is independent of the estimation of the sum field $\sum\mathbf{B}$. The total variance for estimating the gradient is bounded by:

$$
(\delta\nabla B_{x_{\text{est}}})^2+(\delta\nabla B_{y_{\text{est}}})^2+(\delta\nabla B_{z_{\text{est}}})^2 \geq \mathrm{Tr}\left(F_-^{-1}\right)=\frac{4B^2-3B_z^2}{16B^2T^2}+\frac{5B^2+3B_z^2}{16\sin^2(BT)}, \tag{30}
$$

where $B=\sqrt{B_x^2+B_y^2+B_z^2}$. We have verified that the weak commutativity condition (Eq. 8) is satisfied, confirming that a set of POVMs exists which can saturate the quantum Cramér-Rao bound and achieve this precision.

For measurement, we consider local separable measurements on each sensor module ($\mathcal{B}$ and $\mathcal{C}$). Specifically, we choose a projective measurement in the Bell basis for each module:

$$
\Pi_{\mathcal{X}_{00}}=|\Phi^+\rangle\langle\Phi^+|,\quad \Pi_{\mathcal{X}_{01}}=|\Phi^-\rangle\langle\Phi^-|,\quad \Pi_{\mathcal{X}_{10}}=|\Psi^+\rangle\langle\Psi^+|,\quad \Pi_{\mathcal{X}_{11}}=|\Psi^-\rangle\langle\Psi^-|, \tag{31}
$$

where $\mathcal{X}\in\{\mathcal{B},\mathcal{C}\}$ denoted the sensor module and the Bell states are defined in Supplementary Equation 16. This defines 16 measurement operators $\Pi_k=\Pi_{\mathcal{B}i}\otimes\Pi_{\mathcal{C}_j}$ for $i,j\in\{00,01,10,11\}$, which form a complete set ($\sum_k\Pi_k=I$).

The probability of outcome k is $P_k=\langle\Psi_\mathbf{x}|\Pi_k|\Psi_\mathbf{x}\rangle$ for $k\in\{0000,0001,\cdots,1111\}$, where $|\Psi_\mathbf{x}\rangle=U_\mathrm{S}|\Psi_0\rangle$ is the evolved state. For outcomes in the set $\{0011,0111,1011,1100,1101,1110,1111\}$, $P_k=0$ for all parameter values, providing no information. For outcomes in $\{0000,0101,1010\}$, $P_k=0$ specifically at the zero-gradient point ($\nabla\mathbf{B}=(0,0,0)$). Despite these zeros, the probability distribution still encodes information about the parameters.

The classical Fisher information matrix (CFIM) depends on the derivative of P_k with respect

to $x_i, x_j \in \{\nabla B_x, \nabla B_y, \nabla B_z, \sum B_x, \sum B_y, \sum B_z\}$,

$$
\begin{aligned}
[F_C]_{x_i x_j} &= \sum_k \frac{1}{P_k}\left(\frac{\partial P_k}{\partial x_i}\right)\left(\frac{\partial P_k}{\partial x_j}\right) \\
&= \sum_k \frac{\left(\partial x_i \langle \Psi_{\mathbf{x}}|\Pi_k|\Psi_{\mathbf{x}}\rangle\right)\left(\partial x_j \langle \Psi_{\mathbf{x}}|\Pi_k|\Psi_{\mathbf{x}}\rangle\right)}{\langle \Psi_{\mathbf{x}}|\Pi_k|\Psi_{\mathbf{x}}\rangle} \\
&= \sum_k \frac{4\mathrm{Re}\left(\langle \partial x_i \Psi_{\mathbf{x}}|\Pi_k|\Psi_{\mathbf{x}}\rangle\right)\mathrm{Re}\left(\langle \partial x_j \Psi_{\mathbf{x}}|\Pi_k|\Psi_{\mathbf{x}}\rangle\right)}{\langle \Psi_{\mathbf{x}}|\Pi_k|\Psi_{\mathbf{x}}\rangle},
\end{aligned}
\tag{32}
$$

where the sum is over the informative outcomes $k \in \{0000, 0001, 0010, 0100, 0101, 0110, 1000, 1001, 1010\}$. At the zero-gradient point, $\nabla B_x = 0, \nabla B_y = 0$ and $\nabla B_z = 0$, $P_k = \langle \Psi_{\mathbf{x}}|\Pi_k|\Psi_{\mathbf{x}}\rangle = 0$, $\mathrm{Re}\left(\langle \partial x_i \Psi_{\mathbf{x}}|\Pi_k|\Psi_{\mathbf{x}}\rangle\right) = 0$ for $k \in \{0000, 0101, 1010\}$. In these cases, the term is of the form $\frac{0}{0}$ that needs to be calculated through the limit by considering an infinitesimal displacement of $\nabla B_x, \nabla B_y$ and ∇B_z, replacing $|\Psi_{\mathbf{x}}\rangle$ with $|\Psi_{\mathbf{x}}\rangle + \sum_{l=1}^{6} \delta x_l \, |\partial_{x_l} \Psi_{\mathbf{x}}\rangle$. It can then be verified that for all parameters x_i, x_j, $\forall x_i, x_j \in \{\nabla B_x, \nabla B_y, \nabla B_z, \sum B_x, \sum B_y, \sum B_z\}$,

$$
\begin{aligned}
[F_C]_{x_i x_j} = &\sum_{k_1} \frac{\sum_{l_1=1}^{6}\sum_{l_2=1}^{6} 4\delta x_{l_1}\delta x_{l_2} \mathrm{Re}\left(\langle \partial x_i \Psi_{\mathbf{x}}|\Pi_{k_1}|\partial x_{l_1}\Psi_{\mathbf{x}}\rangle\right)\mathrm{Re}\left(\langle \partial x_{l_2} \Psi_{\mathbf{x}}|\Pi_{k_1}|\partial x_j \Psi_{\mathbf{x}}\rangle\right)}{\sum_{l_1=1}^{6}\sum_{l_2=1}^{6} \delta x_{l_1}\delta x_{l_2} \langle \partial x_{l_1}\Psi_{\mathbf{x}}|\Pi_{k_1}|\partial x_{l_2}\Psi_{\mathbf{x}}\rangle} \\
&+ \sum_{k_2} \frac{4\mathrm{Re}\left(\langle \partial x_i \Psi_{\mathbf{x}}|\Pi_{k_2}|\Psi_{\mathbf{x}}\rangle\right)\mathrm{Re}\left(\langle \partial x_j \Psi_{\mathbf{x}}|\Pi_{k_2}|\Psi_{\mathbf{x}}\rangle\right)}{\langle \Psi_{\mathbf{x}}|\Pi_{k_2}|\Psi_{\mathbf{x}}\rangle} \\
= &[F_Q]_{x_i x_j},
\end{aligned}
\tag{33}
$$

where $k_1 \in \{0000, 0101, 1010\}$ and $k_2 \in \{0001, 0010, 0100, 0110, 1000, 1001\}$. The classical Fisher information matrix F_C is thus equal to the quantum Fisher information matrix F_Q. This demonstrates that the QCRB can be saturated by performing local projective measurements in the Bell basis on each sensor module, proving that the precision in Supplementary Equation 30 is achievable.

We also analyze the precision for estimating the gradients of two-dimensional vector fields, namely ∇B_x and ∇B_y, using the same non-local entangled probe state $|\Psi_0\rangle$. The QFIM for estimating $\nabla B_x, \nabla B_y, \sum B_x, \sum B_y$ remains block-diagonal as in Supplementary Equation 25, with the submatrices given by:

$$
F_- = \begin{pmatrix} [F_Q]_{\nabla B_x \nabla B_x} & [F_Q]_{\nabla B_x \nabla B_y} \\ [F_Q]_{\nabla B_x \nabla B_y} & [F_Q]_{\nabla B_y \nabla B_y} \end{pmatrix}, \quad F_+ = \begin{pmatrix} [F_Q]_{\sum B_x \sum B_x} & [F_Q]_{\sum B_x \sum B_y} \\ [F_Q]_{\sum B_x \sum B_y} & [F_Q]_{\sum B_y \sum B_y} \end{pmatrix}, \tag{34}
$$

where

$$
\begin{aligned}
[F_Q]_{\nabla B_x \nabla B_x} &= \frac{4B_x^2 T^2}{B^2} + \frac{B_y^2\left(16\sin^2(BT) - 3\sin^2(2BT)\right)}{B^4}, \\
[F_Q]_{\nabla B_y \nabla B_y} &= \frac{4B_y^2 T^2}{B^2} + \frac{B_x^2\left(16\sin^2(BT) - 3\sin^2(2BT)\right)}{B^4}, \\
[F_Q]_{\nabla B_x \nabla B_y} &= \frac{4B_x B_y T^2}{B^2} - \frac{B_x B_y\left(16\sin^2(BT) - 3\sin^2(2BT)\right)}{B^4}, \\
[F_Q]_{\sum B_x \sum B_x} &= \frac{4B_x^2 T^2}{B^2} + \frac{B_y^2 \sin^2(2BT)}{B^4}, \\
[F_Q]_{\sum B_y \sum B_y} &= \frac{4B_y^2 T^2}{B^2} + \frac{B_x^2 \sin^2(2BT)}{B^4}, \\
[F_Q]_{\sum B_x \sum B_y} &= \frac{4B_x B_y T^2}{B^2} - \frac{B_x B_y \sin^2(2BT)}{B^4},
\end{aligned}
\tag{35}
$$

with $B = \sqrt{B_x^2 + B_y^2}$. The quantum Cramér-Rao bound can also be saturated by performing projective measurement in Bell basis on each sensor module. It can be similarly verified by computing the classical Fisher information matrix using Eq. (3) and comparing it with the quantum Fisher information matrix. The calculation follows a similar procedure to the three-component estimation case and is omitted here for brevity. The total variance for estimating the in-plane gradients can be similarly obtained as

$$\delta\nabla B_{x_{\text{est}}}^2 + \delta\nabla B_{y_{\text{est}}}^2 \geq \frac{1}{4T^2} + \frac{B^2}{4\left(1+3\sin^2(BT)\right)\sin^2(BT)}. \tag{36}$$

a. Adaptive scheme for non-zero gradients. In the experiment, the vector field gradients are measured under the condition $\mathbf{B}_1 = \mathbf{B}_2$, corresponding to zero gradient. However, this can be generalized to arbitrary, non-zero gradients by locally adding a compensation field $\nabla\mathbf{B}_{\text{est}}$ at one sensor node. In each iteration this field is updated, reducing the effective problem to estimating the residual gradient $\nabla\mathbf{B} - \nabla\mathbf{B}_{\text{est}}$, which becomes vanishingly small with the increase of the iteration. Asymptotically, the estimation precision converges to the case with zero gradient.

Figure 1 shows a numerical study of the achievable precision as a function of the residual gradient magnitude. Even for offsets as large as $\|\nabla\mathbf{B} - \nabla\mathbf{B}_{\text{est}}\| = 0.5$, the estimation precision remains close to the ideal case over a sufficiently long evolution time T. The inset in the figure further illustrates how deviations along the X and Y directions affect the estimation performance. In practice, both the compensation field and the evolution time T can be adaptively updated, allowing the protocol to converge asymptotically to the optimal precision. This strategy is conceptually similar to those employed in adaptive quantum metrology [3], where coarse initial estimates are iteratively refined to achieve near-optimal scaling.

1.3.2 Strategy with local entanglement

To estimate the gradients between remote vector fields, a straightforward approach is to first estimate each vector field independently, then calculate the gradient. We first consider the estimation of two gradient components along the x and y directions, $(\nabla B_x, \nabla B_y)$. In this strategy, pairs of locally entangled two-qubit states are used to estimate the local vector fields at two spatially separated sites; the gradient components are subsequently obtained by finite differencing of the two estimates. At each site, the two-dimensional magnetic field is parameterized as $\mathbf{x} = (B, \phi)$, from which the Cartesian components B_x and B_y can be directly inferred [4].

In the experiment, the initial probe state is prepared as the maximally entangled state $|\psi\rangle = \frac{1}{\sqrt{2}}(|00\rangle + |11\rangle)$. Each qubit undergoes the same local unitary evolution $U_s = e^{-iBT\boldsymbol{n}\cdot\boldsymbol{\sigma}}$, resulting in the two-qubit dynamics $U_s \otimes U_s$. A projective measurement in the Bell basis (see Supplementary

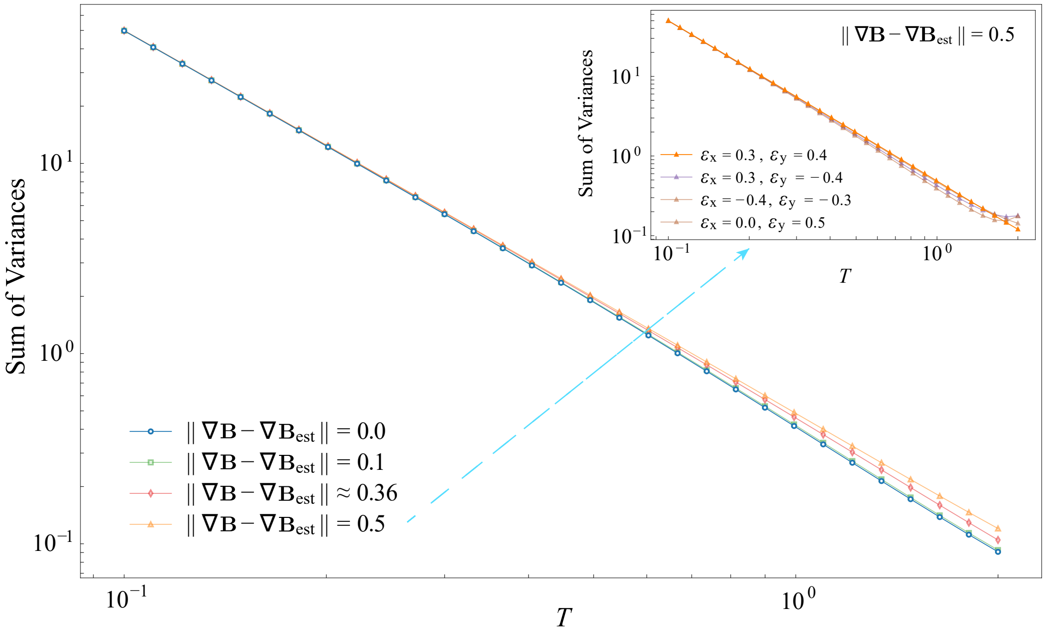

Supplementary Figure 1. **Scaling of sum of variances under different residual gradient norms.** Main Panel: Log–log plot of the total estimation variance for the residual gradient $\nabla\mathbf{B} - \nabla\mathbf{B}_{\rm est}$ as a function of total evolution time T. The true magnetic fields are set as $\mathbf{B}_1 = (\frac{\sqrt{2}}{4}, \frac{\sqrt{2}}{4}, 0)$ and $\mathbf{B}_2 = (0, 0, 0)$, yielding a true gradient $\nabla\mathbf{B} = (\frac{\sqrt{2}}{4}, \frac{\sqrt{2}}{4}, 0)$. A coarse estimate $\nabla\mathbf{B}_{\rm est} = (B_{x\rm est}, B_{y\rm est}, 0)$ is applied at site 2, resulting in a residual gradient $\nabla\mathbf{B} - \nabla\mathbf{B}_{\rm est} = (\epsilon_x, \epsilon_y, 0)$ to be estimated. The four curves correspond to residual norms of $\|\nabla\mathbf{B} - \nabla\mathbf{B}_{\rm est}\| = 0(\epsilon_x = \epsilon_y = 0)$, $\|\nabla\mathbf{B} - \nabla\mathbf{B}_{\rm est}\| = 0.1(\epsilon_x = 0, \epsilon_y = 0.1)$, $\|\nabla\mathbf{B} - \nabla\mathbf{B}_{\rm est}\| \approx 0.36(\epsilon_x = 0.2, \epsilon_y = 0.3)$, and $\|\nabla\mathbf{B} - \nabla\mathbf{B}_{\rm est}\| = 0.5(\epsilon_x = 0.3, \epsilon_y = 0.4)$, respectively. Inset: Scaling behavior of the total estimation variance for different orientations of the residual gradient, with the residual norm $\|\nabla\mathbf{B} - \nabla\mathbf{B}_{\rm est}\|$ fixed at 0.5.

Equation 15) is then performed on the evolved state, yielding the outcome probabilities:

$$
\begin{aligned}
P_{00} &= \left(\cos^2(BT) - \cos(2\phi)\sin^2(BT)\right)^2, \\
P_{01} &= \sin^4(BT)\sin^2(2\phi), \\
P_{10} &= 4\cos^2(BT)\cos^2\phi\sin^2(BT), \\
P_{11} &= 0.
\end{aligned}
\tag{37}
$$

The classical Fisher information matrix for estimating $\mathbf{x} = \{B, \phi\}$ can then be obtained as

$$
F_C = \begin{pmatrix} 16T^2\cos^2\phi & -4T\sin(2BT)\sin(2\phi) \\ -4T\sin(2BT)\sin(2\phi) & 7 - 8\cos(2BT) + 2\cos(4BT)\cos^2\phi - \cos(2\phi) \end{pmatrix}. \tag{38}
$$

This coincides with the QFIM, indicating the measurement saturates the quantum Cramér-Rao bound. Consequently, the variances of the estimators are bounded by

$$
\begin{aligned}
\delta B^2_{\rm est} &= [\mathrm{Cov}(\mathbf{x}_{\rm est})]_{11} \geq [F_C^{-1}]_{11} = \frac{1 - \cos^2(BT)\cos^2\phi}{16T^2\sin^2(BT)\cos^2\phi}, \\
\delta\phi^2_{\rm est} &= [\mathrm{Cov}(\mathbf{x}_{\rm est})]_{22} \geq [F_C^{-1}]_{22} = \frac{1}{16\sin^4(BT)}.
\end{aligned}
\tag{39}
$$

The precision for estimating the two orthogonal components of the vector field using maximally

entangled states is therefore given by

$$\delta B_{x_{\text{est}}}^2 + \delta B_{y_{\text{est}}}^2 = \delta B_{\text{est}}^2 + B^2 \delta\phi_{\text{est}}^2 \geq \frac{1}{16\sin^2(BT)}\left(\frac{B^2 - \cos^2(BT)B_x^2}{T^2 B_x^2} + \frac{B^2}{\sin^2(BT)}\right). \tag{40}$$

Gradients are estimated by differencing the independent estimates from two sites, which introduces an overall factor of two in the summed gradient precision:

$$\delta\nabla B_{x_{\text{est}}}^2 + \delta\nabla B_{y_{\text{est}}}^2 \geq \frac{1}{8\sin^2(BT)}\left(\frac{B^2 - \cos^2(BT)B_x^2}{T^2 B_x^2} + \frac{B^2}{\sin^2(BT)}\right). \tag{41}$$

While the maximally entangled state is used experimentally for its ease of implementation, the precision within this local-entanglement strategy can be further improved by optimizing the input probe state. For a fair comparison, we now present the optimal probe and its corresponding theoretical lower bound, which follows the analysis in [4]. The optimal initial probe state in this case is given by

$$|\psi_{opt}\rangle = \frac{1}{\sqrt{2}}(U_r \otimes U_r)(|11\rangle - |00\rangle), \tag{42}$$

where $U_r = e^{i\frac{BT}{2}\boldsymbol{n}\cdot\boldsymbol{\sigma}} e^{-i\frac{\phi}{2}\sigma_z} e^{-i\frac{\pi}{4}\sigma_y}$. This optimal state depends on the true values of the unknown parameters B and ϕ. As they are not known a priori, an adaptive strategy needs to be adopted in practice by replacing the true values with their current estimates, B_{est} and ϕ_{est}. This yields the practical preparation unitary $U_r^{\text{est}} = e^{i\frac{B_{\text{est}}T}{2}\boldsymbol{n}\cdot\boldsymbol{\sigma}} e^{-i\frac{\phi_{\text{est}}}{2}\sigma_z} e^{-i\frac{\pi}{4}\sigma_y}$. In the asymptotical limit when the estimates converge to the true value, we obtain the highest precision as

$$\delta B_{x_{\text{est}}}^2 + \delta B_{y_{\text{est}}}^2 = \delta B_{\text{est}}^2 + B^2 \delta\phi_{\text{est}}^2 \geq \frac{1}{4}\left(\frac{1}{\langle\Delta^2 G_B\rangle} + \frac{B^2}{\langle\Delta^2 G_\phi\rangle}\right) \geq \frac{1}{16T^2} + \frac{B^2}{16\sin^2(BT)}. \tag{43}$$

This bound is achievable by performing projective measurement in the Bell basis. Gradients are again estimated by differencing the independent estimates from two sites, which introduces an overall factor of two in the summed gradient precision:

$$\delta\nabla B_{x_{\text{est}}}^2 + \delta\nabla B_{y_{\text{est}}}^2 \geq \frac{1}{8T^2} + \frac{B^2}{8\sin^2(BT)}. \tag{44}$$

We then consider the estimation of gradients of three dimensional fields, $(\nabla B_x, \nabla B_y, \nabla B_z)$. Similarly, pairs of locally entangled two-qubit states are used to estimate the local vector fields at two sites; the gradient components are subsequently obtained by finite differencing of the two estimates. At each site, the two-dimensional magnetic field is parameterized as $\mathbf{x} = (B, \theta, \phi)$, from which the Cartesian components can be obtained.

To ensure a fair comparison, we again take the maximally entangled probe state $|\psi\rangle = \frac{1}{\sqrt{2}}(|00\rangle + |11\rangle)$. Each qubit undergoes the same local unitary evolution $U_s = e^{-iBT\boldsymbol{n}\cdot\boldsymbol{\sigma}}$, resulting in the two-qubit dynamics $U_s \otimes U_s$. The QFIM for estimating $\mathbf{x} = \{B, \theta, \phi\}$ is given by

$$F_Q = \begin{pmatrix} [F_Q]_{BB} & [F_Q]_{B\theta} & [F_Q]_{B\phi} \\ [F_Q]_{B\theta} & [F_Q]_{\theta\theta} & [F_Q]_{\theta\phi} \\ [F_Q]_{B\phi} & [F_Q]_{\theta\phi} & [F_Q]_{\phi\phi} \end{pmatrix} \tag{45}$$

with

$$
\begin{aligned}
[F_Q]_{BB} =& 4T^2 \left(3+\cos(2\theta)+2\cos(2\phi)\sin^2\theta\right) , \\
[F_Q]_{\theta\theta} =& 2\sin^2(BT) \left(3+3\cos(2BT)\cos(2\phi)+2\sin^2\phi+\cos^2(BT)\left(2-4\cos(2\theta)\sin^2\phi\right)\right. \\
&\left.+4\cos\theta\sin(2BT)\sin(2\phi)\right) , \\
[F_Q]_{\phi\phi} =& 2\sin^2(BT)\sin^2\theta \left(2+2\sin^2(BT)+2\sin^2\theta+2\sin^2\phi+\cos(2BT)\left(\cos(2\theta)-3\cos(2\phi)\right)\right. \\
&\left.+2\sin^2(BT)\cos(2\theta)\cos(2\phi)-4\sin(2BT)\cos\theta\sin(2\phi)\right) , \\
[F_Q]_{B\theta} =& 4T\left(2\sin^2(BT)\sin\theta\sin(2\phi)-\sin(2BT)\sin(2\theta)\sin^2\phi\right) , \\
[F_Q]_{B\phi} =& -16T\sin(BT)\sin^2\theta\sin\phi\left(\cos(BT)\cos\phi+\sin(BT)\cos\theta\sin\phi\right) , \\
[F_Q]_{\theta\phi} =& 2\sin^2(BT)\left(\sin(2BT)\sin\theta\left((3+\cos 2\theta)\cos(2\phi)+2\sin^2\theta\right)-2\cos(2BT)\sin(2\theta)\sin(2\phi)\right) .
\end{aligned}
\tag{46}
$$

The resulting QFIM is singular, implying that, for the maximally entangled probe state considered here, the three parameters are not simultaneously identifiable. Consequently, within this local-entanglement strategy based on the maximally entangled state, a direct three-parameter benchmark analogous to the two-parameter case is not available.

1.3.3 Additional strategy and comparison

In addition to the two strategies for gradient sensing discussed above-the distributed strategy with non-local entanglement (NLE) and the local strategy with local entanglement (LE)—we also consider an alternative strategy based on the remote sensing (RS) described in the first section. For this strategy, the remote magnetic fields at each location are estimated independently, with the central module serving as an ancilla, and the gradient is calculated from the separate field estimates. The experimental circuits for these three strategies are illustrated in Supplementary Figure 2. For a more comprehensive comparison, we also present the precision of gradient estimation achievable with this alternative strategy. The comparison is made under the condition that the resource allocation is identical, with each sensor module containing two sensors. In table 1, we list the precision achieved by various strategies for estimating gradients along x and y directions. Supplementary Figure 3 further compares their performance by quantifying the estimation precision under various conditions. Specifically, Supplementary Figure 3**d** identifies the parameter regime where NLE outperforms RS and LE, while Supplementary Figures 3**e–g** present systematic comparisons of precision as functions of B, T, and the cycle numbers N.

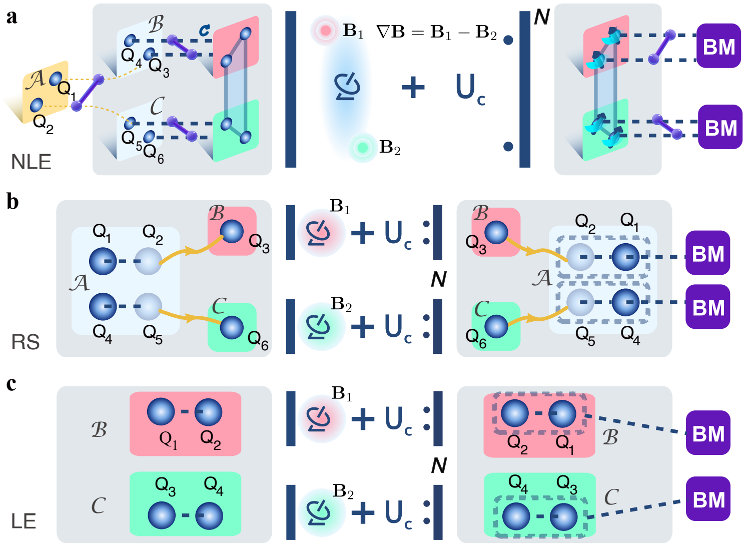

Supplementary Figure 2. **The sequences for measuring the gradient by NLE strategy, and separately measuring vector fields $\mathbf{B}_1$ and $\mathbf{B}_2$, and calculating their difference (RS/LE strategy).** **a,** Non-local entanglement strategy with three modules, where Q_3, Q_4 on $\mathcal{B}$ and Q_5, Q_6 on $\mathcal{C}$ serve as the sensor qubits. **b,** Remote sensing strategy with modules $\mathcal{A}-\mathcal{B}$ and $\mathcal{A}-\mathcal{C}$, where Q_3 on $\mathcal{B}$ and Q_6 on $\mathcal{C}$ act as the sensor qubits, Q_1 and Q_4 on $\mathcal{A}$ serve as the ancilla qubits. **c,** Local entanglement strategy with modules $\mathcal{B}$ and $\mathcal{C}$, where $\mathbf{B}_1$ is applied on Q_1 and Q_2, $\mathbf{B}_2$ is applied on Q_3 and Q_4.

**Strategy**	**Initial state**	**Precision**	**Optimality**	**Achievability**
**NLE**	$\frac{1}{\sqrt{2}}(\lvert 0011\rangle - \lvert 1100\rangle)$	$\frac{1}{4T^2} + \frac{B^2}{4(1+3\sin^2(BT))\sin^2(BT)}$	N	Y
**RS**	$\frac{1}{\sqrt{2}}(\lvert 00\rangle + \lvert 11\rangle)$	$\frac{1}{4T^2} + \frac{B^2}{4\sin^2(BT)}$	Y	Y
**LE**	$\frac{1}{\sqrt{2}}\left(\lvert +_B\rangle^{\otimes 2} - \lvert -_B\rangle^{\otimes 2}\right)$	$\frac{1}{8T^2} + \frac{B^2}{8\sin^2(BT)}$	Y	Y
	$\frac{1}{\sqrt{2}}(\lvert 00\rangle + \lvert 11\rangle)$	$\frac{1}{8\sin^2(BT)}\left(\frac{B^2-\cos^2(BT)B_x^2}{T^2B_x^2} + \frac{B^2}{\sin^2(BT)}\right)$	N	Y

Supplementary Table 1. The precision for estimating gradients along x and y directions.

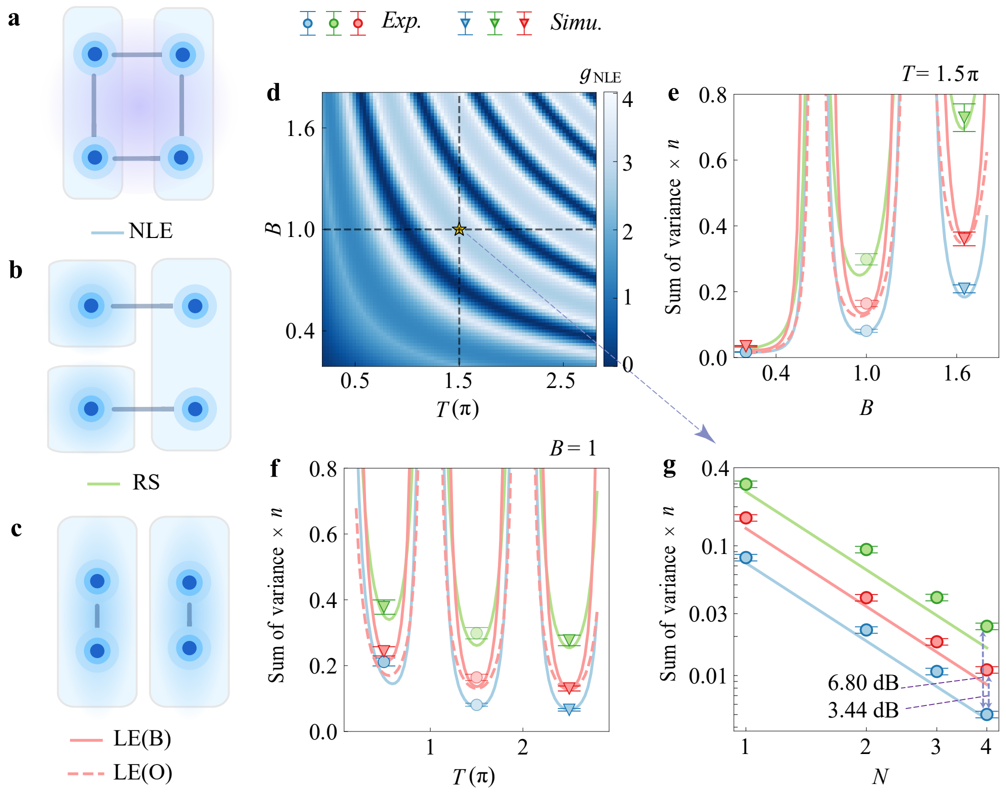

Supplementary Figure 3. **Strategies comparison for gradient estimation of a 2-component vector field. a-c,** Schematic diagrams of different strategies: (**a**) Distributed sensing with non-local entanglement (NLE); (**b**) Remote sensing (RS) with an ancilla qubit; (**c**) Sensing with local entanglement (LE). **d,** Parameter range where NLE outperforms RS and LE. The minimum precision gain of NLE over RS and LE(B) across different B and T values, is calculated by their theoretical precision. **e-f,** Comparison of the precision ($\sum_{i\in\{x,y\}} \delta^2 \nabla B_{i_{\text{est}}}$) of the three strategies. (**e**) Precision versus B for the three strategies at $T = 1.5\pi$ and $N = 1$. (**f**) Impact of T on estimation precision at $B = 1$ and $N = 1$. (**g**) Estimation precision versus N for the three strategies at $T = 1.5\pi$ and $B = 1$. The solid and dashed curves: the theoretical precision bound. LE(B): local entanglement strategy using Bell state as the probe state, and Bell measurement. LE(O): local entanglement strategy using the optimal probe state and measurement. The definition of the error bars in (e)-(g) is described in Methods.

Supplementary Note 2 – Experimental implementation

2.1 Device information

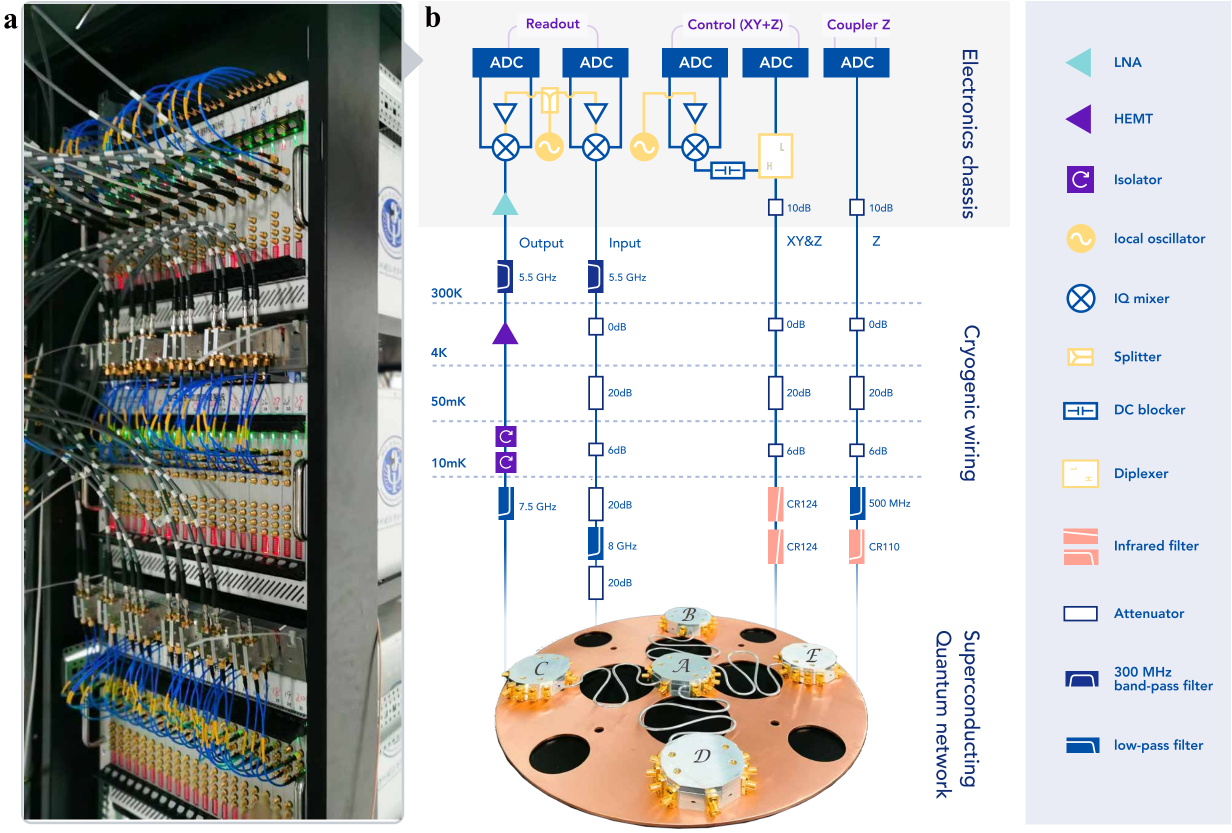

Supplementary Figure 4. **The experimental setup.** **a:** The microwave control and measurement system built for this experiment. **b:** The schematic diagram of the room-temperature electronics chassis, cryogenic wiring, and superconducting quantum network inside the dilution refrigerator. Right panel: the legend of the devices.

We implement the distributed quantum metrology experiment utilizing a modular quantum computing platform composed of five superconducting quantum chips, each integrated with four qubits. The inter-chip connectivity is facilitated by high-quality aluminum superconducting coaxial cables, where a gmon coupler is positioned between the qubits engaged in communication and the cable, enabling tunable coupling strength. Furthermore, an impedance transformer is designed on chip to significantly mitigate the stray loss on the communication channels [5]. In Fig. 4, we show the comprehensive structure of the experimental setup. The distributed quantum processors are sheltered in the 10 mK environment, nestled beneath the mixing chamber of a dilution refrigerator. The microwave cables connecting the superconducting quantum chips serve as the conduit for signal transmission and reception between the quantum processors (see the bottom part of the middle

panel) and the customized integrated electronic channels (see the left photograph). The electronics is primarily composed of digital-to-analog converters (DAC) and analog-to-digital converters (ADC), which orchestrate the generation, manipulation and readout of quantum control signals. The generation of XY control signals (single-qubit rotation) is facilitated by IQ mixing of the MHz output of the DACs and the GHz microwave carrier from a local oscillator (LO). Concurrently, the Z control signals (qubit frequency modulation) originate from DC and pulse signals output of the DACs. The XY signals and Z signals belonging to each qubit are combined with a customized diplexer in room temperature. The readout pulses are generated by another set of DACs, LO and IQ mixers, these devices up-convert the probe photons to match the readout resonator frequencies, conversely, the emitted photonic signals from the readout resonators are amplified and down-converted, finally being sampled by the ADCs, completing the readout cycle and providing a digital record of the measurement data. For higher control and readout quality, we deploy multiple filters across different temperature zones within the experimental setup (see the middle panel), the legends detailing the components of these stages are shown in the adjacent panel on the right.

**Node**	$\mathcal{A}$		$\mathcal{B}$		$\mathcal{C}$	
**Qubit**	Q_1	Q_2	Q_3	Q_4	Q_5	Q_6
$\omega_{\text{idle}}/2\pi$ (GHz)	4.551	5.019	4.477	4.959	4.937	4.393
$\omega_{\text{read}}/2\pi$ (GHz)	5.629	5.692	5.686	5.627	5.688	5.621
$E_C/2\pi$ (MHz)	−212	−200	−210	−225	−210	−229
F_{00}	0.94	0.94	0.88	0.86	0.92	0.90
F_{11}	0.91	0.90	0.87	0.83	0.88	0.89
T_1 (μs)	19.2	18.4	26.8	14.3	25.3	26.2
T_{2R} (μs)	1.52	4.77	2.42	4.72	3.58	4.02
T_{2E} (μs)	5.49	10.95	11.49	15.18	15.57	14.45
**SQG RB fid(%)**	99.96	99.91	99.95	99.47	99.80	99.75
**CZ XEB fid(%)**	98.50		97.30		98.40	

Supplementary Table 2. Device information

The experiment in this work involves three distributed quantum processors, each containing two qubits. We list the basic information of these six qubits in Table 2. All qubits are designed to be operated across a frequency range of $4.1 \sim 5.1$ GHz, and idled at staggered frequencies. The resonator frequencies are also staggered for independent readout. The anharmonicity E_C is a parameter determined by the capacitance of each qubit, it is instrumental in shaping the interaction essential for the construction of Controlled-Z (CZ) gates. F_{00} and F_{11} are the state preparation and measurement (SPAM) fidelity for $|0\rangle$ and $|1\rangle$. T_1 parameter denotes the energy relaxation time of each qubit, T_{2R} and T_{2E} represent the dephasing time characterized by Ramsey experiment and spin echo experiment, respectively.

2.2 Gate performance

In our experiment, we calibrate and benchmark both local two-qubit gates (CZ, CNOT) and inter-module state transfer operations, together with single-qubit gate performance, in order to establish the reliable entanglement generation and well-characterized metrological gate set.

The CZ gates were optimized using a standard calibration protocol [6], with a pulse consisting of a 36 ns plateau and 10 ns flattop rising and falling edges. We benchmark the gate performance using cross-entropy benchmarking (XEB) [7], obtaining an average CZ gate fidelity of 98.1%.

To enable quantum state transfer across modules, we employ a vacuum Rabi protocol between the communication qubit and the inter-chip cable. This synchronizes the qubit frequencies on both sides of the cable by tuning them into resonance. Simultaneously, the coupler pulses are optimized to achieve the desired effective coupling strength while minimizing reflection losses [5]. The fidelity of the transferred state, reconstructed by quantum state tomography (QST), reaches an average of $\sim 98.4\%$.

The control-signal sequence is implemented by gate sets in the U(3) formalism: $U_3(\alpha, \beta, \lambda) = R_z(\beta)R_x(\pi/2)R_z(\alpha)R_x(-\pi/2)R_z(\lambda)$. The gate set consists of three virtual Z rotations (λ, α, β) interleaved with an $X/2$ gate and a $-X/2$ gate. The single qubit gates (SQG) are benchmarked by randomized benchmarking (RB) experiment, yielding an averaged fidelity of 99.81%.

2.3 Gate set calibration

To further ensure accurate multi-qubit control, we carefully calibrate the phases associated with entangling gates. An ideal CZ gate applies only a π phase to the $|11\rangle$ state, while leaving other computational basis states unchanged. In practice, however, frequency detuning between the two qubits leads to additional single-qubit phases on $|01\rangle$ and $|10\rangle$, which manifest as relative $U(1)$ rotations in the two-qubit rotating frame. We characterize these phases using Ramsey sequences, and compensate them with appropriate virtual-Z corrections. As shown in Supplementary Figure 5**a**, by repeating layers of phase-compensated CZ gates, we observe oscillatory interference fringes between $|0\rangle$ and $|1\rangle$ states, allowing us to identify the phase setting that ensures constructive alignment as the number of two-qubit gate layers increases (Supplementary Figure 5**d**).

Similar phase accumulation effects arise not only in CZ gates but also in CNOT and remote state transfer operations. We quantify and compensate these phases using Ramsey-type sequences (Fig. 5 **a-c**), which reveal increasing sensitivity with the number of repeated operations. By sweeping the compensation phase, we obtain population profiles that converge to stable values (Fig. 5 **d-f**), from which the optimal corrections can be extracted. These corrections are then consistently applied across the full gate set, ensuring that all local and non-local operations remained phase-aligned.

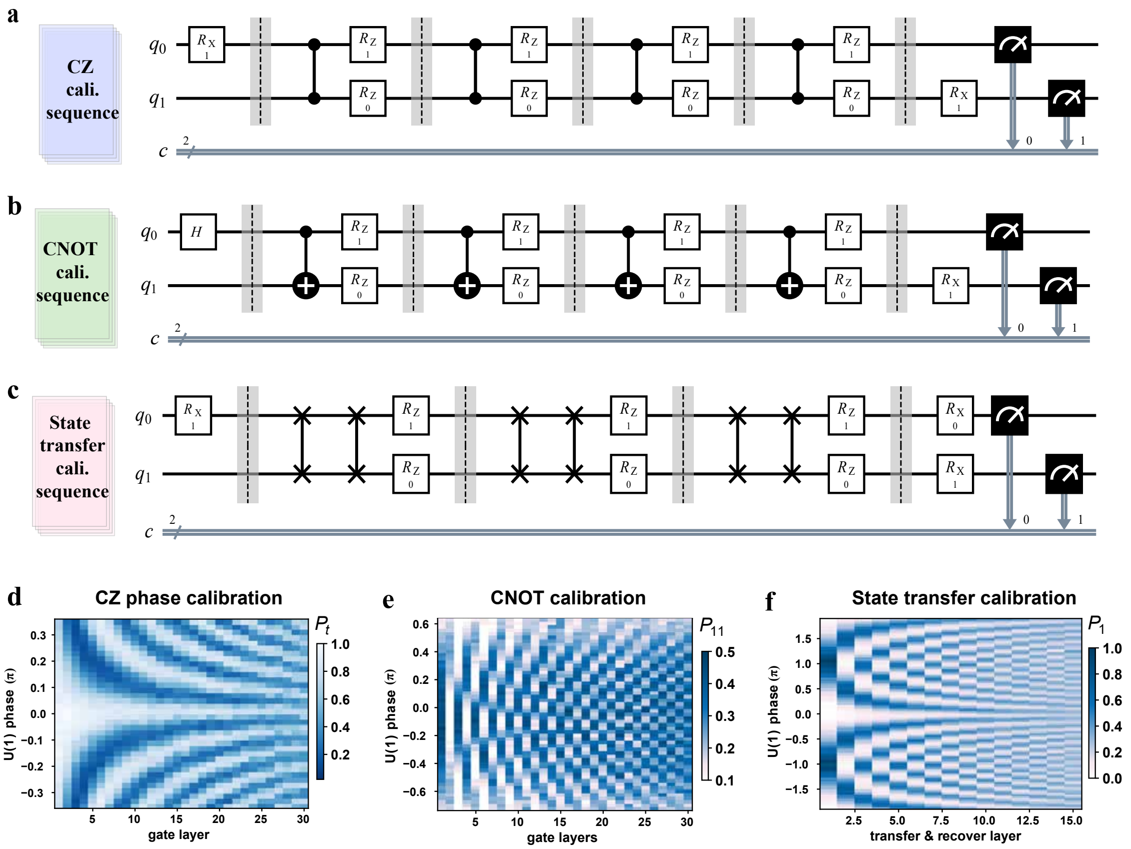

Supplementary Figure 5. **Repeated gate sequences (drawn by Qiskit [8]) for $U(1)$ phase calibration.** **a,** CZ phase calibration sequence. The virtual Z phase of the control qubit is fixed, while that of the target qubit is swept, and the resulting probability on the target qubit (P_t) is measured to extract the accumulated phase. **b,** CNOT phase calibration sequence. A CNOT gate is compiled into a $-Y/2$ gate, followed by a CZ gate and a $Y/2$ gate. The control qubit is initialized in the $|+\rangle$ state by a Hadamard gate, and repeated CNOT operations reveal oscillations between $\frac{1}{\sqrt{2}}(|00\rangle + |11\rangle)$ and $|00\rangle$, with the correct compensating phase maximizing the exchange, exhibited by the probability on state $|11\rangle$ (P_{11}). **c,** Calibration sequence for inter-module quantum state transfer. We transfer a $|0\rangle + i|1\rangle$ state from the transmission qubit (q_0) to the receiving qubit (q_1), and then use another state transfer to recover it back to the transmission qubit. This transfer and recover set is repeated by multiple layers, and the result is measured on X-axis of q_1. **d–f,** Extraction of virtual Z phases from repeated sequences of CZ, CNOT gates, and state transfer operations, respectively.

2.4 Implementation of distributed sensing in quantum circuits

The NLE strategy for gradient metrology is implemented using the three-node sensor network illustrated in Fig. 6, composed of modules $\mathcal{A}$, $\mathcal{B}$, and $\mathcal{C}$ (Fig. 6**a**). As illustrated in Fig. 6**b**, the protocol begins by generating a Bell pair between Q_1 and Q_2 on module $\mathcal{A}$. Subsequently, the two qubits of this pair are transferred simultaneously: one from Q_1 to Q_3 on module $\mathcal{B}$, and the other from Q_2 to Q_5 on module $\mathcal{C}$. CNOT gates are then applied on both modules $\mathcal{B}$ and $\mathcal{C}$, resulting in a GHZ state across the two modules with a fidelity of 80.36% (Fig. 6**c**). The probe state $|\Psi_0\rangle$ is prepared by applying additional X gates on Q_3 and Q_4, and a Z gate on Q_5, achieving a fidelity

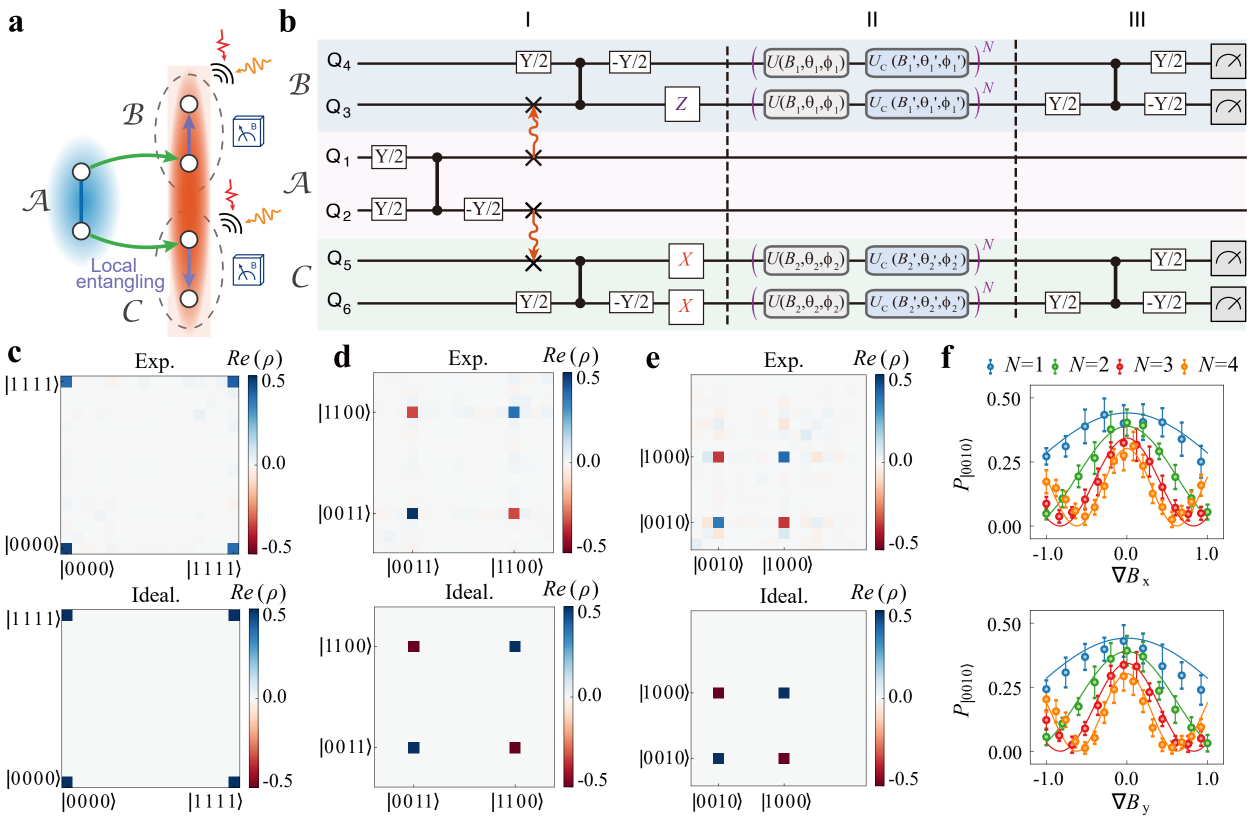

Supplementary Figure 6. **a,** A brief schematic diagram of NLE metrology strategy for gradient. **b,** The detailed quantum circuit of (a). **c,** The remote four-qubit GHZ state generated across three modules. **d,** The four-qubit probe state $|\Psi_0\rangle = \frac{1}{\sqrt{2}}(|0011\rangle - |1100\rangle)$. **e,** The reference final state $|\Psi_f\rangle = \frac{1}{\sqrt{2}}(|0010\rangle - |1000\rangle)$ after step III, with no control and signal units inserted into the circuit. **f,** The probability oscillation observed when scanning parameter ∇B_x or ∇B_y for different N, the encoding time is fixed at $T = 0.2\pi$. The error bars denote the standard deviation.

of 76.16% (see Fig. 6**d**). Subsequently, we encode the spatially distributed vector field on sensor chips $\mathcal{B}$ and $\mathcal{C}$ with $U(3)$-formalism gate sets, where $\mathbf{B}_1$ acts on both Q_3 and Q_4, $\mathbf{B}_2$ acts on both Q_5 and Q_6. Following the encoding process, we conduct Bell measurement on both modules $\mathcal{B}$ and $\mathcal{C}$. Under optimal control, the ideal final state $|\Psi_f\rangle$ has equivalent occupation on $|0010\rangle$ and $|1000\rangle$, the fidelity obtained from the experiment is 75.20% (see Fig. 6**e**). The information carried by the sensors is decoded into the probability distribution in the measurement basis (see Fig. 6**f** for P_{0010}). The oscillation period of this probability with respect to the gradient components ∇B_x and ∇B_y is observed to contract as the number of sequential copies N increases.

The error in this sequence primarily stems from the control error when synchronously transferring two entangled states. The control error is estimated to be 11.44% for generating the non-local GHZ state; the decoherence error throughout this 340 ns sequence is approximately 8.34%. These two parts yield an estimated fidelity of 80.22%, which is close to our experimental result of 80.36%. The non-local entangled state across two chips, which are not directly connected, is more fragile to environmental noise. The effective decoherence rate of the probe state is estimated to be

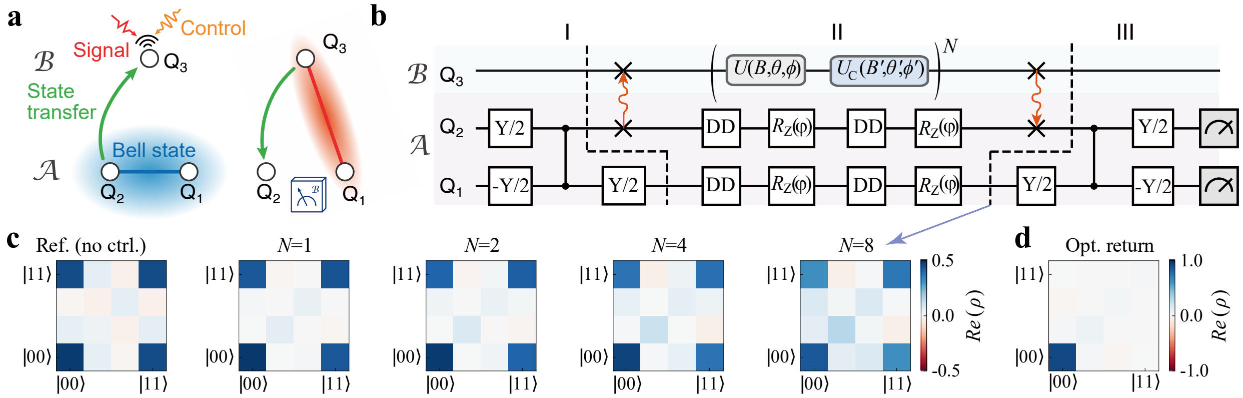

Supplementary Figure 7. **a,** A brief schematic diagram for sensing a local vector field at a certain position with two nodes $\mathcal{A}$ and $\mathcal{B}$. **b,** The quantum circuit diagram for **a**. I: state preparation, II: encoding, III: measurement. **c,** Ref (no ctrl): the density matrix with no signal-control sequence, measured after step II. $N = 1 \sim 4$: the density matrices with $N = 1, 2, 4, 8$, measured after step II. **d,** Opt return: the density matrix with no signal-control sequence, measured after step III.

$80 \times 2\pi$ kHz. As a consequence, the fidelity values of $|\Psi_f\rangle$ are $69.27\%, 63.81\%, 58.78\%, 54.15\%$ for $N = 1 \sim 4$, respectively. These values are higher than the confidence threshold $\sim 50\%$ for entanglement.

Demonstrating the remote sensing strategy requires pairs of distributed nodes with high-quality connections. Using the $\mathcal{A}$-$\mathcal{B}$ pair as an example (Fig. 1**a** in the main text), a locally generated entangled state (blue shadow) is transferred to the remote node $\mathcal{B}$, establishing cross-module entanglement (red shadow). This setup forms the basis of our conceived metrology network: a central module ($\mathcal{A}$) is connected to multiple remote sensor modules ($\mathcal{B}, \mathcal{C}$, etc.), which are spatially positioned to perform distributed sensing of a local field.

The quantum circuit is illustrated in Fig. 7**b**. We first generate a local Bell state on chip $\mathcal{A}$, and subsequently transfer the state of one qubit, Q_2 on chip $\mathcal{A}$ to Q_3 on module $\mathcal{B}$, resulting in a cross-node Bell state between Q_1 and Q_3. Q_3 is then used as the sensor qubit and Q_1 as the ancillary qubit. To protect the inter-module entanglement from fast decoherence during the sensing interval, we apply dynamical decoupling sequences to Q_1 and Q_2. The quantum state is subsequently transferred from the sensor qubit (Q_3) back to Q_2, followed by a Bell measurement on Q_1, Q_2. Under optimal control, the entire Step II functions as an identity operation. We perform quantum state tomography on module $\mathcal{A}$ after state retrieval. Figure 7**c** displays the extracted real parts of the density matrices for Q_2 and Q_3.

To capture errors from state preparation and measurement (SPAM), we implement a reference circuit. The reference circuit is obtained from the original ciruit by excluding signal encoding, control operations, dynamical decoupling, and phase compensation (R_z gates), i.e., only keeps the state preparation and measurement. The reference circuit achieves a fidelity of 91.15% for the preparation of the probe state, and after including additional gates required for Bell-basis measurement, it

yields a fidelity of 90.52%. In contrast, circuits incorporating signal encoding and optimal control sequences—with dynamical decoupling and R_z gates for phase compensation—were evaluated for $N = 1, 2, 4, 8$ repetitions of the signal-control unit. The corresponding state fidelities are 88.13%, 84.37%, 79.02%, and 70.46%, respectively. The decline in fidelity with increasing N reflects error accumulation during the sensing protocol. By comparing these results with the reference fidelity, we can separate SPAM errors from those introduced by the signal-control sequence. The total error per signal-control unit is estimated at approximately 3.34%, comprising an average control error of 0.79% per U_x or U_c operation and a decoherence contribution of 2.55%, the latter corresponding to an effective decoherence rate of $41.1 \times 2\pi$ kHz.

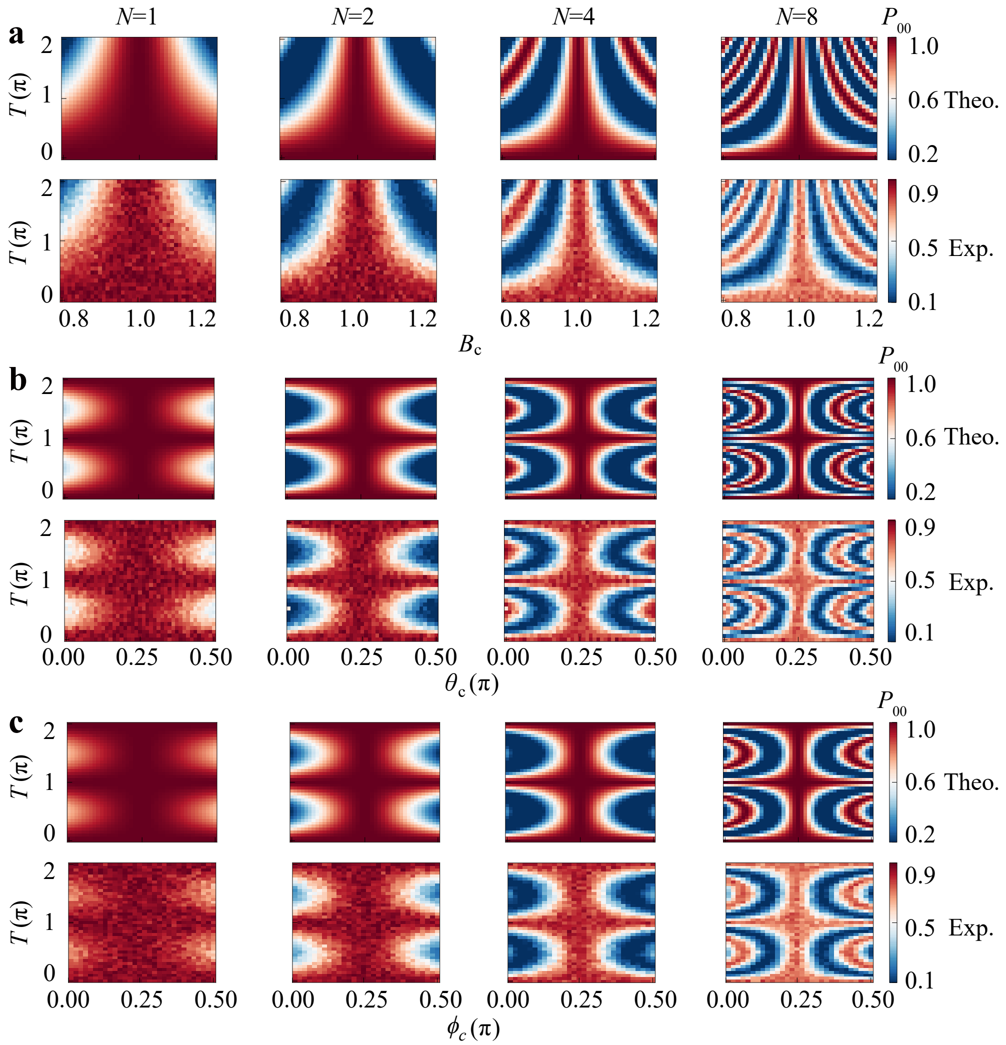

Supplementary Figure 8. **The probability oscillation with three parameters and encoding time T. a,** For a fixed signal, we scan control parameter B_c and encoding time T for different N. **b,** We scan control parameter θ_c and encoding time T for different N. **c,** We scan control parameter ϕ_c and encoding time T for different N.

A key feature of the relationship between the number of control-signal layers (N) and the

estimated precision is evident in the oscillation period of the probability derived from Bell measurement. As shown in Supplementary Figure 8, the period of the P_{00} profile near the optimal control parameters is inversely proportional to N. Scanning a single parameter is analogous to a single-parameter estimation process. In this context, the quantum Fisher information is determined by the derivative of the probability with respect to the target parameter. The reduction in oscillation period with increasing N thus provides an intuitive understanding of the enhancement offered by the sequential strategy. Moreover, the encoding time T is also periodically correlated to the probability distribution, and this correlation depends on the signal parameters. For example, when the signal is set to $(B, \theta, \phi) = (1, \pi/4, \pi/4)$, the period of P_{00} profile at $T = \pi, 2\pi$ stays invariant with respect to the parameters θ and ϕ, regardless of N. In contrast, at $T = 0.5\pi$ or $T = 1.5\pi$, the oscillation period changes more dramatically, indicating that the sequential strategy is effective there.

To implement LE strategy, we generate local Bell state on node $\mathcal{B}$ and $\mathcal{C}$, signals and controls are simultaneously acting on two pairs of sensor qubits. The sequence ends with Bell measurement on $\mathcal{B}$ and $\mathcal{C}$, which is similar to RS strategy.

Supplementary Note 3 – Extended data

3.1 Extended data for sensing of remote vector fields

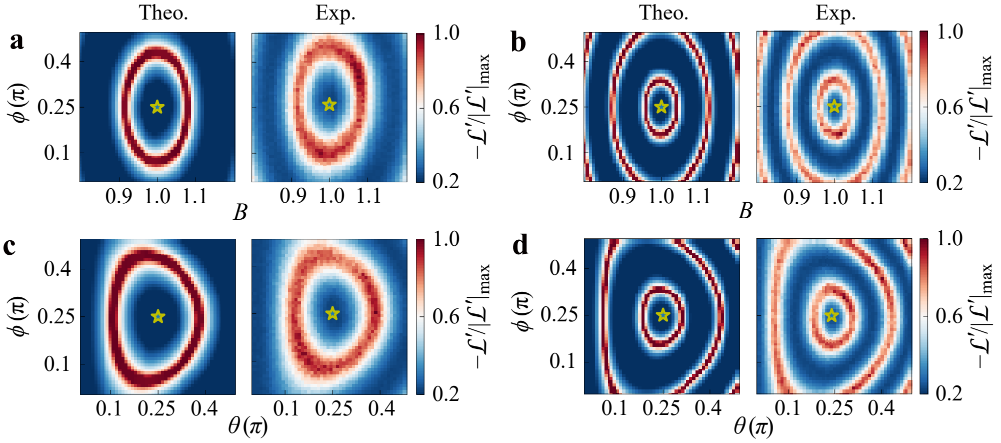

Supplementary Figure 9. **The likelihood function landscape at** $N = 4$ **and** $N = 8$**.** Stars: the location of the optimal control parameters. **a,** The landscape for parameter B and ϕ at $N = 4$. **b,** The landscape for parameter B and ϕ at $N = 8$. **c,** The landscape for parameter θ and ϕ at $N = 4$. **d,** The landscape for parameter θ and ϕ at $N = 8$. We post the theoretical landscape and experimental result, and mark the optimal control parameters.

We benchmark the sensor-ancilla network by analyzing the landscape of the likelihood function $\mathcal{L}'$ near the optimal control parameters, the results are shown over two variables in Supplementary Figure 9. Specifically, the panels depict $\mathcal{L}'(B, \phi)$ in Supplementary Figures 9**a,b** and $\mathcal{L}'(\theta, \phi)$ in Supplementary Figures 9**c,d**. The optimal control parameters, marked with a star in each panel, correspond to the expected estimation results. As N increases, the boundary area of the likelihood landscape contracts, demonstrated for $N = 4$ in Supplementary Figures 9**a,c** and $N = 8$ in Supplementary Figures 9**b,d**. The agreement between theoretical and experimental results ensures that the estimated parameters not only align with the observed data but also adhere to the underlying physical model. Moreover, in a qualitative perspective, the slight blurring and distortion on the contour of the experimental landscape, comparing to the ideal results, reflect the impact of decoherence and control errors on the protocol, respectively.

The Supplementary Figure 10 illustrates the complete MLE result for sensing the three-component vector field. The density amplitude in each panel represents the count of the distribution normalized by the integral of the distribution. These results demonstrate that increasing the number of sequential copies enhances the precision of simultaneous three-parameter estimation. However, unavoidable experimental errors introduce a bias of up to ±3.45% in the averaged estimation results, deviating from the actual vector field parameters. These errors can distort the likelihood landscape, affecting the efficiency of the MLE process. Moreover, fluctuation in exper-

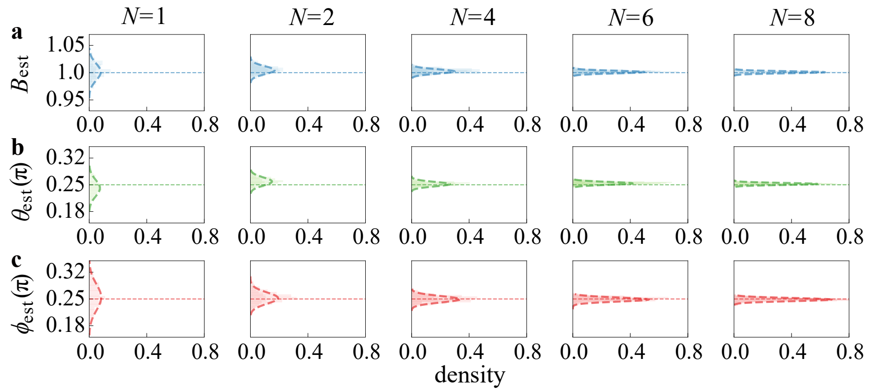

Supplementary Figure 10. **The result for simultaneously estimating three parameters of a local vector field $\mathbf{B}(B, \theta, \phi)$.** Bars: MLE result histograms. Dashed curves: gaussian fitting of the histograms. Dashed lines: the ideal signal parameters. **a,** The density distribution of parameter B. **b,** The density distribution of parameter θ. **c,** The density distribution of parameter ϕ.

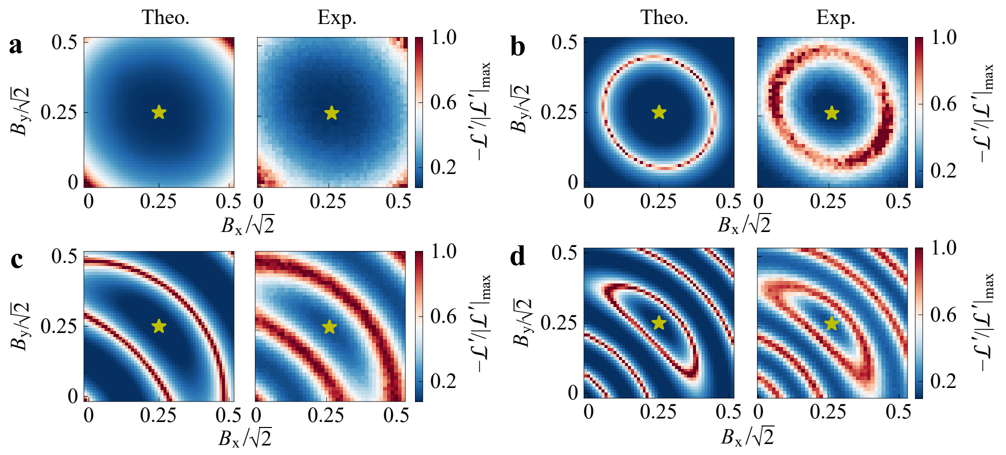

Supplementary Figure 11. **The theoretical and experimental landscape of estimating a two-component vector field with a sensor-ancilla network.** Stars: the location of the optimal control parameters. **a,** At $N = 2$ and $T = 0.5\pi$. **b,** At $N = 4$ and $T = 0.5\pi$. **c,** At $N = 2$ and $T = 1.5\pi$. **d,** At $N = 4$ and $T = 1.5\pi$

imental noise lead to inhomogeneous probability distributions, increasing the risk of convergence to local rather than global minima. Despite these challenges, the optimal strategy we implement facilitates a flat region around the optimal control parameters, and the use of multiple initial values mitigates the impact of local minima [9]. This approach improves the robustness and reliability of the estimation process [2].

We apply the same benchmarking approach to a two-component vector field. As shown in Supplementary Figure 11, the contraction of the likelihood function landscape with increasing N is evident. The parameters to be estimated in this case are set as $\mathbf{B} = (\frac{\sqrt{2}}{2}, \frac{\sqrt{2}}{2}, 0)$, corresponding to $|\mathbf{B}| = 0.5$.

3.2 Extended data for distributed sensing of vector field gradient

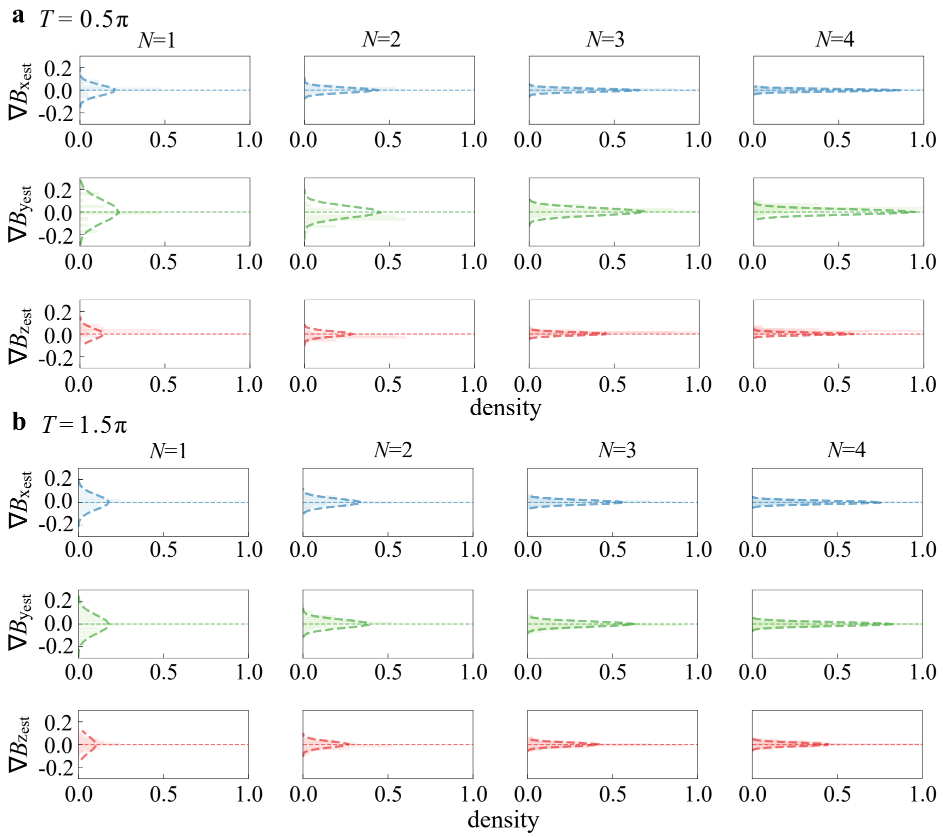

Supplementary Figure 12. **The normalized distribution of estimators for simultaneously three-component estimation.** Bars: MLE result histograms. Dashed curves: gaussian fitting of the histograms. Dashed lines: the ideal signal parameters. **a,** At $T = 0.5\pi$ and $N = 1 \sim 4$. **b,** At $T = 1.5\pi$ and $N = 1 \sim 4$.

We experimentally evaluate the performance of NLE strategy for simultaneously estimating the three components of the gradient. In this scheme, we set $\mathbf{B} = (\frac{1}{2}, \frac{1}{2}, \frac{\sqrt{2}}{2})$, the normalized distributions of the estimators $\nabla B_{x_{\mathrm{est}}}$, $\nabla B_{y_{\mathrm{est}}}$, and $\nabla B_{z_{\mathrm{est}}}$ are shown in Supplementary Figure 12**a** (for $T = 0.5\pi$) and Supplementary Figure 12**b** (for $T = 1.5\pi$).

When estimating the gradient of a two-component vector field $\nabla\mathbf{B} = (\nabla B_x, \nabla B_y)$, the field amplitudes are expressed as a function of gradient $\nabla\mathbf{B}$ and sum $\sum\mathbf{B}$ at two distinct positions, with $\mathbf{B}_1 = (\sum\mathbf{B} + \nabla\mathbf{B})/2$ and $\mathbf{B}_2 = (\sum\mathbf{B} - \nabla\mathbf{B})/2$. The full dataset, presented in Fig. 2 of the main text, is shown in Supplementary Figure 13, where the x and y components are plotted separately. The signal parameters are chosen as $\sum\mathbf{B} = (\sqrt{2}/2, \sqrt{2}/2, 0)$ and $\nabla\mathbf{B} = (0, 0, 0)$. The encoding times are set to $T = 0.5\pi$ (Supplementary Figure 13**a**) and $T = 1.5\pi$ (Supplementary Figure 13**b**).

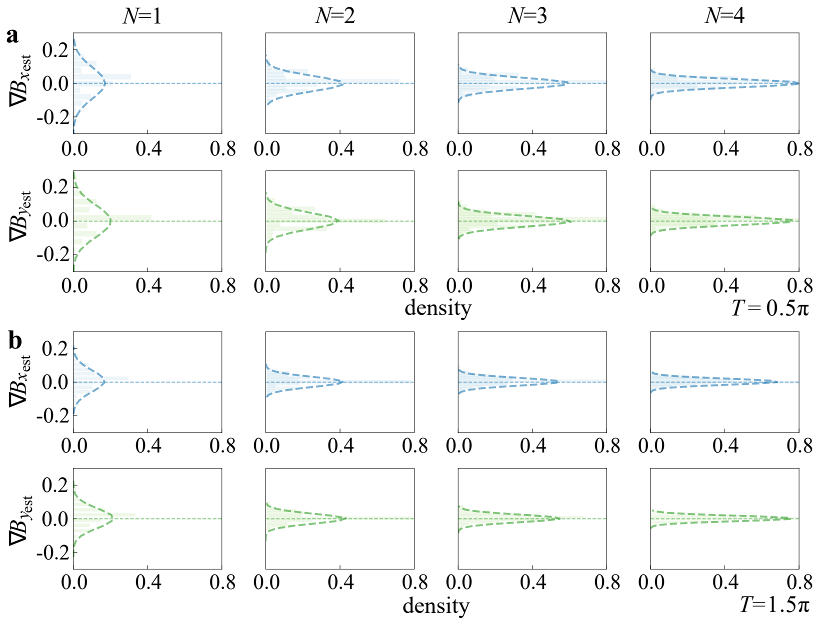

Supplementary Figure 13. **The density distribution for estimated x and y components of the vector field gradient.** Bars: MLE result histograms. Dashed curves: gaussian fitting of the histograms. Dashed lines: the ideal signal parameters. **a,** The density at $T = 0.5\pi$ for $N = 1, 2, 3, 4$. **b,** The density at $T = 1.5\pi$ for $N = 1, 2, 3, 4$.

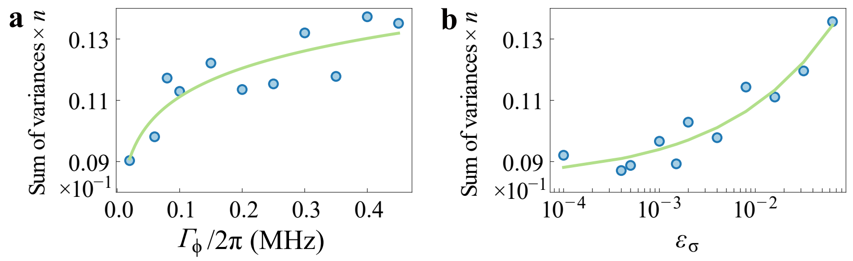

Supplementary Figure 14. **The influence of circuit error on the MLE result.** Dots: simulation data. Solid curves: fitting result. **a** The relation between simulated precision and dephasing rate. **b** The relation between simulated precision and gate error.

3.3 The influence of noise

Different types of noise in quantum system have impact to the precision of the gradiometer. The effects of noisy channels in quantum parameter estimation have been discussed in previous studies [10–15]. In our work, the dominant sources of noise are control errors and dephasing. We numerically simulate the quantitative relationship between these noise types and the precision of the estimation. Supplementary Figure 14**a** shows the sum of variances in the estimated gradient as a function of different dephasing rates Γ_ϕ, while Supplementary Figure 14**b** depicts the effect of varying gate errors ϵ_σ on the precision. The dephasing noise is modeled using a thermal channel, and gate errors are incorporated through a Pauli noise channel. These simulations are conducted using the Qiskit framework [8].

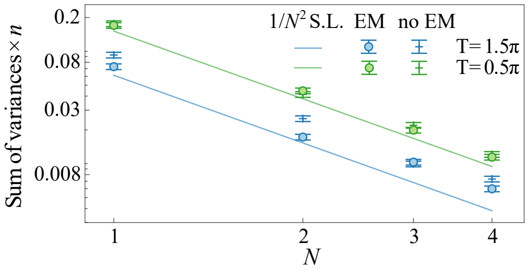

Supplementary Figure 15. **The effect of error mitigation (EM) on the experimental sum of variances.** Different markers indicate data obtained with EM and without EM. The definition of the error bars is described in Methods.

To mitigate the effects of noise, we apply error mitigation (EM) techniques [16] during data post-processing. For the NLE strategy, non-local entangled states are particularly sensitive to environmental noise. However, EM significantly improves performance, as demonstrated in Supplementary Figure 15.

[1] Yuan, H. Sequential feedback scheme outperforms the parallel scheme for hamiltonian parameter estimation. *Phys. Rev. Lett.* **117**, 160801 (2016).

[2] Hou, Z. *et al.* Zero–trade-off multiparameter quantum estimation via simultaneously saturating multiple heisenberg uncertainty relations. *Sci. Adv.* **7**, eabd2986 (2021).

[3] Pang, S. & Jordan, A. N. Optimal adaptive control for quantum metrology with time-dependent hamiltonians. *Nat. Commun.* **8**, 14695 (2017).

[4] Hou, Z. *et al.* Minimal tradeoff and ultimate precision limit of multiparameter quantum magnetometry under the parallel scheme. *Phys. Rev. Lett.* **125**, 020501 (2020).

[5] Niu, J. *et al.* Low-loss interconnects for modular superconducting quantum processors. *Nat. Electron.* **6**, 235–241 (2023).

[6] Sung, Y. *et al.* Realization of high-fidelity cz and zz-free iswap gates with a tunable coupler. *Phys. Rev. X* **11**, 021058 (2021).

[7] Goss, N. *et al.* High-fidelity qutrit entangling gates for superconducting circuits. *Nat. Commun.* **13** (2022).

[8] Javadi-Abhari, A. *et al.* Quantum computing with qiskit (2024). arXiv:2405.08810.

[9] Kuroda, M., Mori, Y. & Iizuka, M. *Initial Value Selection for the Alternating Least Squares Algorithm*, 227–239 (Springer Singapore, 2020).

[10] Le, T. K., Nguyen, H. Q. & Ho, L. B. Variational quantum metrology for multiparameter estimation under dephasing noise. *Sci. Rep.* **13** (2023).

[11] Yuan, H. & Fung, C.-H. F. Quantum parameter estimation with general dynamics. *npj Quantum Inf.* (2017).

[12] Escher, B. M., de Matos Filho, R. L. & Davidovich, L. General framework for estimating the ultimate precision limit in noisy quantum-enhanced metrology. *Nat. Phys.* **7**, 406–411 (2011).

[13] Wang, K. *et al.* Entanglement-enhanced quantum metrology in a noisy environment. *Phys. Rev. A* **97**, 042112 (2018).

[14] Chaves, R., Brask, J. B., Markiewicz, M., Kołodyński, J. & Acín, A. Noisy metrology beyond the standard quantum limit. *Phys. Rev. Lett.* **111**, 120401 (2013).

[15] Peng, J.-X., Zhu, B., Zhang, W. & Zhang, K. Enhanced quantum metrology with non-phase-covariant noise. *Phys. Rev. Lett.* **133**, 090801 (2024).

[16] Conlon, L. O. *et al.* Approaching optimal entangling collective measurements on quantum computing platforms. *Nat. Phys.* **19**, 351–357 (2023).
